# The differing pathophysiologies that underlie COVID‐19‐associated perniosis and thrombotic retiform purpura: a case series

**DOI:** 10.1111/bjd.19415

**Published:** 2021-01-01

**Authors:** C.M. Magro, J.J. Mulvey, J. Laurence, S. Sanders, A.N. Crowson, M. Grossman, J. Harp, G. Nuovo

**Affiliations:** Department of Pathology and Laboratory Medicine Weill Cornell Medicine New York NY USA; Department of Laboratory Medicine Memorial Sloan Kettering Cancer Center New York NY USA; Department of Medicine Division of Hematology and Medical Oncology Weill Cornell Medicine New York NY USA; Sanders Dermatology New City NY USA; Regional Medical Laboratories Pathology Laboratory Associates and University of Oklahoma Tulsa OK USA; Department of Dermatology Yale University New Haven, CT and Hofstra/Northwell New Hyde NY USA; Department of Dermatology Weill Cornell Medicine New York NY USA; The Ohio State University Comprehensive Cancer CenterColumbus Ohio and Discovery Life Sciences Powell OH USA

## Abstract

**Background:**

There are two distinctive acral manifestations of COVID‐19 embodying disparate clinical phenotypes. One is perniosis occurring in mildly symptomatic patients, typically children and young adults; the second is the thrombotic retiform purpura of critically ill adults with COVID‐19.

**Objectives:**

To compare the clinical and pathological profiles of these two different cutaneous manifestations of COVID‐19.

**Methods:**

We compared the light microscopic, phenotypic, cytokine and SARS‐CoV‐2 protein and RNA profiles of COVID‐19‐associated perniosis with that of thrombotic retiform purpura in critical patients with COVID‐19.

**Results:**

Biopsies of COVID‐19‐associated perniosis exhibited vasocentric and eccrinotropic T‐cell‐ and monocyte‐derived CD11c^+^, CD14^+^ and CD123^+^ dendritic cell infiltrates. Both COVID‐associated and idiopathic perniosis showed striking expression of the type I interferon‐inducible myxovirus resistance protein A (MXA), an established marker for type I interferon signalling in tissue. SARS‐CoV‐2 RNA, interleukin‐6 and caspase 3 were minimally expressed and confined to mononuclear inflammatory cells. The biopsies from livedo/retiform purpura showed pauci‐inflammatory vascular thrombosis without any MXA decoration. Blood vessels exhibited extensive complement deposition with endothelial cell localization of SARS‐CoV‐2 protein, interleukin‐6 and caspase 3; SARS‐CoV‐2 RNA was not seen.

**Conclusions:**

COVID‐19‐associated perniosis represents a virally triggered exaggerated immune reaction with significant type I interferon signaling. This is important to SARS‐CoV‐2 eradication and has implications in regards to a more generalized highly inflammatory response. We hypothesize that in the thrombotic retiform purpura of critically ill patients with COVID‐19, the vascular thrombosis in the skin and other organ systems is associated with a minimal interferon response. This allows excessive viral replication with release of viral proteins that localize to extrapulmonary endothelium and trigger extensive complement activation.

The severe acute respiratory distress syndrome‐associated coronavirus‐2 (SARS‐CoV‐2), the aetiological agent of coronavirus disease 2019 (COVID‐19), was identified in Wuhan, Hubei, China in December 2019 and declared a pandemic by the World Health Organization in early March 2020. Its higher infectivity and lower mortality rates differentiate it from SARS‐CoV.^1^–^3^ Most patients with COVID‐19 have a self‐limited illness. However, there are predisposed populations, including the elderly and patients with certain pre‐existing conditions such as diabetes, hypertension and obesity, in whom higher mortality rates have been observed.^4^

We recently reported that a complement‐driven pauci‐inflammatory thrombotic form of septal capillary injury causes severe progressive acute respiratory distress syndrome (ARDS) in the setting of COVID‐19.^5^ We showed that patients critically ill with COVID‐19 have widespread systemic complement activation destroying microvasculature and exacerbating concurrent thrombophilia, resulting in organ dysfunction attributable to vascular thrombosis. This type of complement‐driven thrombotic syndrome is rarely encountered as a complication of COVID‐19 in the paediatric setting, where the clinical course is typically banal.^6^ However, uncommonly, children have developed perniosis or a multisystemic inflammatory syndrome resembling Kawasaki disease during the COVID‐19 pandemic and presumably in response to SARS‐CoV‐2 infection.^7^–^12^

The skin is an easily accessible window to understand the pathophysiological basis of COVID‐19 and its heterogeneous clinical phenotype. One pole of the cutaneous spectrum is the perniosis‐like acral dermatitis occurring predominantly in children and less commonly in adults, who present with no or mild additional symptoms of viral infection such as cough or low‐grade fever. For the purposes of this paper we will designate this eruption as ‘COVID‐19‐associated perniosis’, defining it as a form of virally triggered perniosis to distinguish it from cold‐induced (‘idiopathic’) perniosis.^13^ The opposite pole, COVID‐19‐associated thrombotic retiform purpura, is a cutaneous extension of the severe microangiopathic ARDS seen in the critically ill.^5^ The hallmarks are a pauci‐inflammatory thrombogenic vasculopathy associated with activation of the mannan‐binding lectin and alternative complement pathways.

We describe three cases of COVID‐19‐associated perniosis and compare them with six cases of idiopathic perniosis and six cases of thrombotic retiform purpura seen in critically ill patients with COVID‐19 from a clinical, light microscopic, immunophenotypic, viral and molecular perspective. We propose that COVID‐19‐associated perniosis represents a virally induced archetypical interferonopathy, reflective of the robust immune response typical of children and mechanistically distinct from the interferon‐poor complement‐driven retiform purpura encountered in very sick patients with COVID‐19.^13^

## Materials and methods

Samples of COVID‐19‐associated perniosis were sectioned at a thickness of 5 µm and stained on positively charged glass slides stored at 4 °C within 3 days after sectioning. Deparaffinization, rehydration and antigen retrieval were performed on the BOND‐III Leica automated slide stainer (Leica Biosystems, Newcastle upon Tyne, UK) following the methodologies that have been previously described.^14^, ^15^ Specimens were incubated and variably stained with CD3 (LN10), CD4 (4B12), CD8 (4B11), CD7 (LP15), CD11c (5D11), CD20, myeloperoxidase, and granzyme (11F1) from Leica Biosystems; lysozyme (Rabbit Poly) from Agilent (Santa Clara, CA, USA); and myxovirus virus resistance protein A (MXA, C‐1) from Santa Cruz Biotechnology (Santa Cruz, CA, USA) at room temperature, followed by visualization with the Leica BOND detection kit for 20 min at room temperature.^16^ The specimens were then counterstained with haematoxylin and coverslipped.^14^, ^15^ For the cases of thrombotic retiform purpura, immunohistochemical staining of C3d (ready‐to‐use polyclonal antibody; Cell Marque, Rocklin, CA, USA), C4d (1: 50, monoclonal antibody; Quidel, San Diego, CA, USA), mannan‐binding lectin serine peptidase (MASP)‐2 (Sigma, St Louis, MO, USA; HPA029313) and C5b‐9 (1: 250, clone aE11; Dako, Glostrup, Denmark) was accomplished using the BOND‐III autostainer.

Further viral studies were performed on the cases of COVID‐19‐associated perniosis and COVID‐19 thrombotic retiform purpura and three cases of perniosis unrelated to COVID‐19. Positive and negative controls were used with every run, including autopsy lung tissue from patients with COVID‐19 pneumonia and normal lung tissue obtained prior to 2019, respectively. Antibodies specific for the envelope protein (1: 300) and spike protein (1: 7000) of COVID‐19 (ProSci, Poway, CA, USA) were optimized with the lung tissues. Optimal pretreatment conditions included ethylenediaminetetraacetic acid antigen retrieval solution (pH 9·0) for 30 min. The analyses were done on the automated Leica BOND platform, with the modification that the Enzo Life Sciences (Farmingdale, CA, USA) peroxidase antimouse/rabbit conjugate (catalogue #ADI‐950‐113‐0100) was used in place of the equivalent Leica conjugate as this reduced background.

Detection of SARS‐CoV‐2 RNA was done using the ACD RNAscope probe (catalogue #848561‐C3; ACD, Newark, CA, USA) as per a previously published protocol in which only the viral RNA probe is changed. The SARS‐CoV‐2 probe targets the target region nt21631–23303 derived from the genomic sequence of the virus (NC_045512·2). In brief, pretreatment in the ACD RNA retrieval solution and protease digestion is followed by overnight hybridization at 37 °C and detection using the ACD 2·5 High Definition diaminobenzidine detection kit.

## Results

### COVID‐19‐associated perniosis

#### Case 1

**Figure 1:**
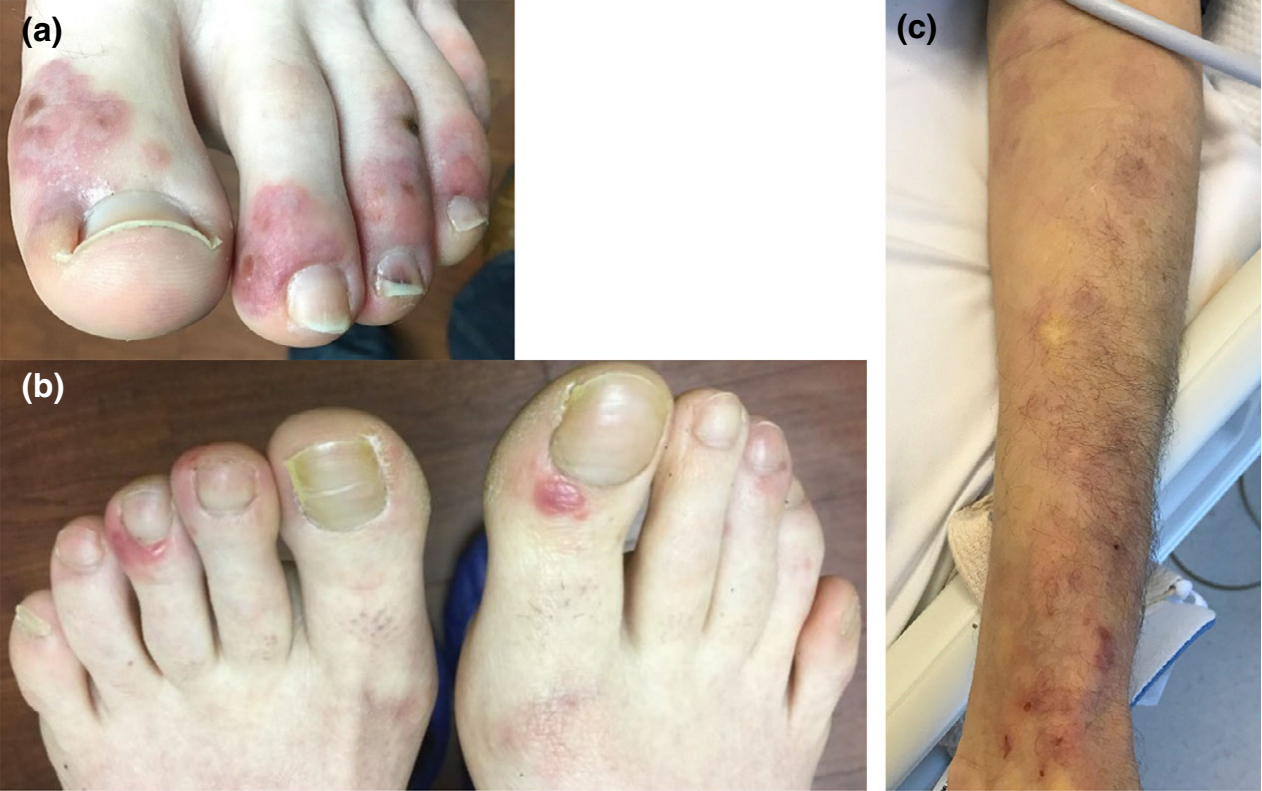
Clinical morphology of COVID‐19‐associated perniosis, idiopathic perniosis and COVID‐19‐associated thrombotic retiform purpura. (a) In early April of 2020, the patient, a 16‐year‐old New York city resident, developed bilateral perniotic‐like skin lesions without cold exposure. They are more extensive than conventional perniosis and include areas of ulceration and focal targetoid areas. (b) This 36‐year‐old woman presents with discrete somewhat painful erythematous papules on the distal toes temporally associated with cold exposure, compatible with idiopathic perniosis. (c) This critically ill patient with severe COVID‐19 demonstrates a mottled livedoid rash of the arms bilaterally.

A 16‐year‐old boy developed bilateral painful perniotic‐like plaques on his toes (Figure 1a). He had been confined to his home and denied any significant cold exposure. His brother had a cough and fever several weeks earlier, which resolved without viral diagnostic testing. A nasopharyngeal swab was negative for SARS‐CoV‐2. Serologies to assess for past SARS‐CoV‐2 infection were not performed.

#### Case 2

A 48‐year‐old female resident of New City, New York, developed fever, mild leucocytosis, a purpuric macular skin rash of the legs and perniotic lesions on the toes. A skin biopsy was performed on one of the toe lesions. Her nasopharyngeal swab test for COVID‐19 was negative. Her COVID‐19‐associated IgG and IgM serologies were also negative.

#### Case 3

The patient was a 65‐year‐old woman who lived in very close proximity to the South Dakota Smithfield Pork processing plant, which had experienced a COVID‐19 outbreak affecting 700 of its 3000 employees. At the time of the outbreak she developed distinct papular lesions of the fingers accompanied by a cough. Over the ensuing weeks the lesions underwent full resolution. She declined COVID‐19 testing.

#### Light microscopic findings and phenotypic studies

**Figure 2:**
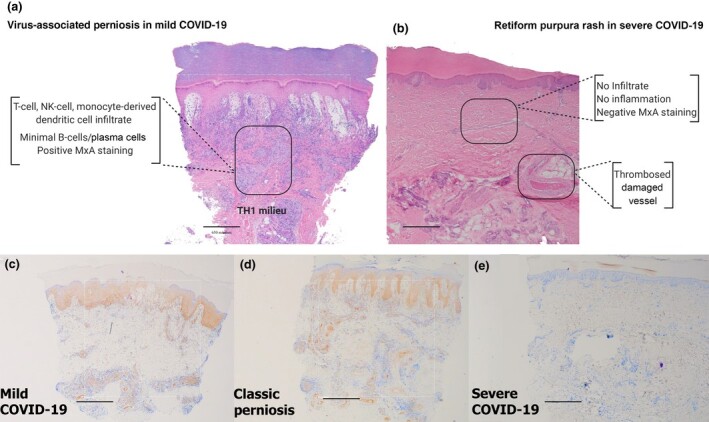
Comparative light microscopic findings and myxovirus resistance protein A (MXA) pattern of immunoreactivity in COVID‐19‐associated perniosis, idiopathic perniosis and COVID‐19‐associated thrombotic retiform purpura. (a) In acral perniosis of mild COVID‐19, a striking inflammatory response is observed characterized by a mononuclear‐cell‐dominant interface dermatitis with an associated intense superficial and deep angiocentric lymphocytic and histiocytic infiltrate that surrounds and infiltrates the vessel walls of capillaries, venules and arterioles and is found in close apposition to the eccrine ducts and glands of the eccrine coil [haematoxylin and eosin (HE), original magnification × 40]. (b) In contrast, with the livedoid rash of severe COVID‐19 there is a pauci‐inflammatory thrombogenic vasculopathy that in this case involves the arterial system (HE, × 40). (c) In COVID‐19‐associated perniosis there is immunoreactivity for MXA within the epidermis and endothelial cells and amid inflammatory cells [diaminobenzidine (DAB), × 40]. (d) A virtually identical pattern of immunoreactivity is observed for MXA in idiopathic perniosis (DAB, × 40). (e) In the paucicellular thrombotic retiform purpura of severe COVID‐19 there is no immunoreactivity for MXA (DAB, × 40). NK, natural killer; TH, T helper cell. Scale bars are set at 650 microns.

**Figure 3:**
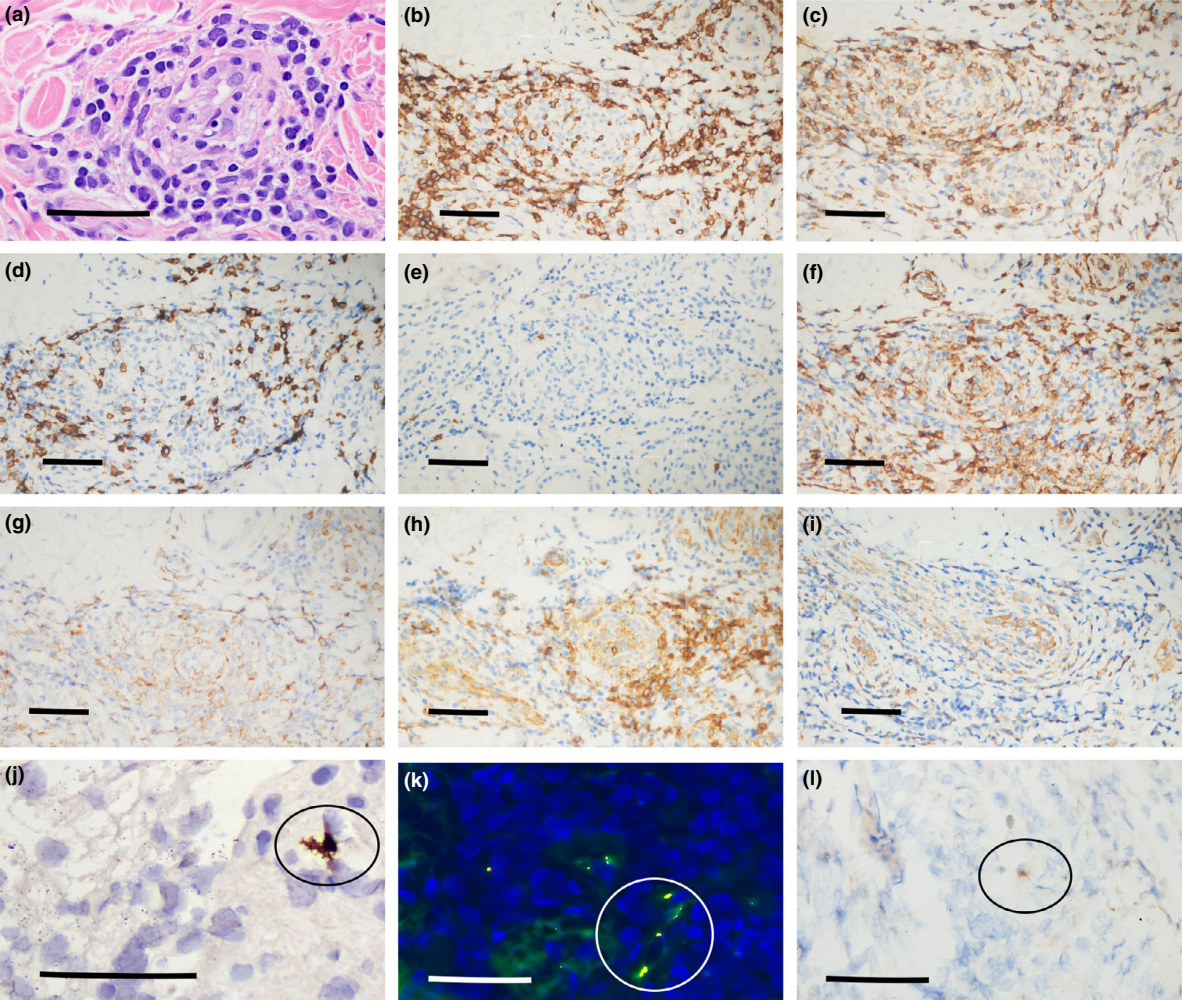
Light microscopic, inflammatory cell phenotypic profile and SARS‐CoV‐2 viral protein distribution in COVID‐19‐associated perniosis. (a) There is a brisk angiocentric lymphocytic and histiocytic infiltrate. While the infiltrate permeates the vessels and endothelial cell swelling is observed there is no frank fibrin deposition (haematoxylin and eosin, original magnification × 400). (b) The infiltrate is predominated by CD3^+^ T cells [diaminobenzidine (DAB), × 200]. (c) The CD4 antibody highlights T cells and histiocytes (DAB, × 200). (d) A minor lymphoid populace is represented by CD8 (DAB, × 200). (e) The CD20 antibody shows only rare B cells (DAB, × 200). (f–h) The histiocytes demonstrate positivity for CD14 (f, DAB, × 200) CD11c (g, DAB, × 200) and CD123 (h, DAB, × 200). (i) There is positive immunoreactivity for myxovirus resistance protein A within the epidermis, endothelia and inflammatory cells in all cases (DAB, × 200). (j) In all three cases of COVID‐19‐associated perniosis, only rare SARS‐CoV‐2 RNA‐positive cells are present (one to three cells) (DAB, × 1000). (k) Antibody to SARS‐CoV‐2 envelope protein (DAB) is colocalized with SARS‐CoV‐2 membrane (red chromogen). Using Nuance software (PerkinElmer, Waltham, MA, USA) the envelope protein fluoresces green while the membrane protein fluoresces red, producing a yellow signal when colocalized in a few extravascular mononuclear cells, likely of histiocytic derivation (DAB, × 400). (l) In the two studied cases of perniosis only a few to rare positive cells for interleukin‐6 are observed and are primarily mononuclear cells likely of histiocytic origin (DAB, × 400). Scale bars are set at 125 microns.

The cases showed a similar morphology characterized by a mononuclear‐cell‐dominant interface dermatitis with an associated dense superficial and deep angiocentric lymphocytic and histiocytic infiltrate that surrounded and infiltrated the blood vessels and was adjacent to the eccrine coil, ducts and glands (Figures 2a and 3a). The histiocytes demonstrated reniform and serpiginous nuclei and intracytoplasmic cellular debris (Figure 3a). Papillary dermal oedema was seen in cases 1 and 3 (Figure 2a). There was endothelial cell swelling and red cell extravasation. Fibrin deposition within reticular dermal‐based blood vessels was present but minimal.

Phenotypic studies revealed an infiltrate predominated by CD3^+^ T cells (Figure 3b) and CD163^+^ CD68^+^ histiocytes. Rare CD20‐positive B cells were seen (Figure 3e). The CD4 to CD8 ratio was within normal limits (Figure 3c, d). The histiocytes were highlighted by CD163, lysozyme, CD68, CD14 (Figure 3f), CD11c (Figure 3g) and CD123 (Figure 3h); a subset was positive for myeloperoxidase. Significant MXA positivity in the epidermis, endothelia and inflammatory cells was seen in the three cases (Figures 2c and 3i). Complement studies, namely C3d, C4d and C5b‐9, were conducted and found to be either absent or minimally positive.

### Idiopathic perniosis

Six cases of idiopathic perniosis were retrieved from the archival files of the Division of Dermatopathology, Weill Cornell College of Medicine. The biopsies were procured from three male and three female patients ranging in age from 16 to 65 years. A diagnosis was made of idiopathic perniosis in all patients. The typical clinical presentation is illustrated in Figure 1(b) and is characterized by discrete erythematous papules on the toes and/or fingers.

**Figure 4:**
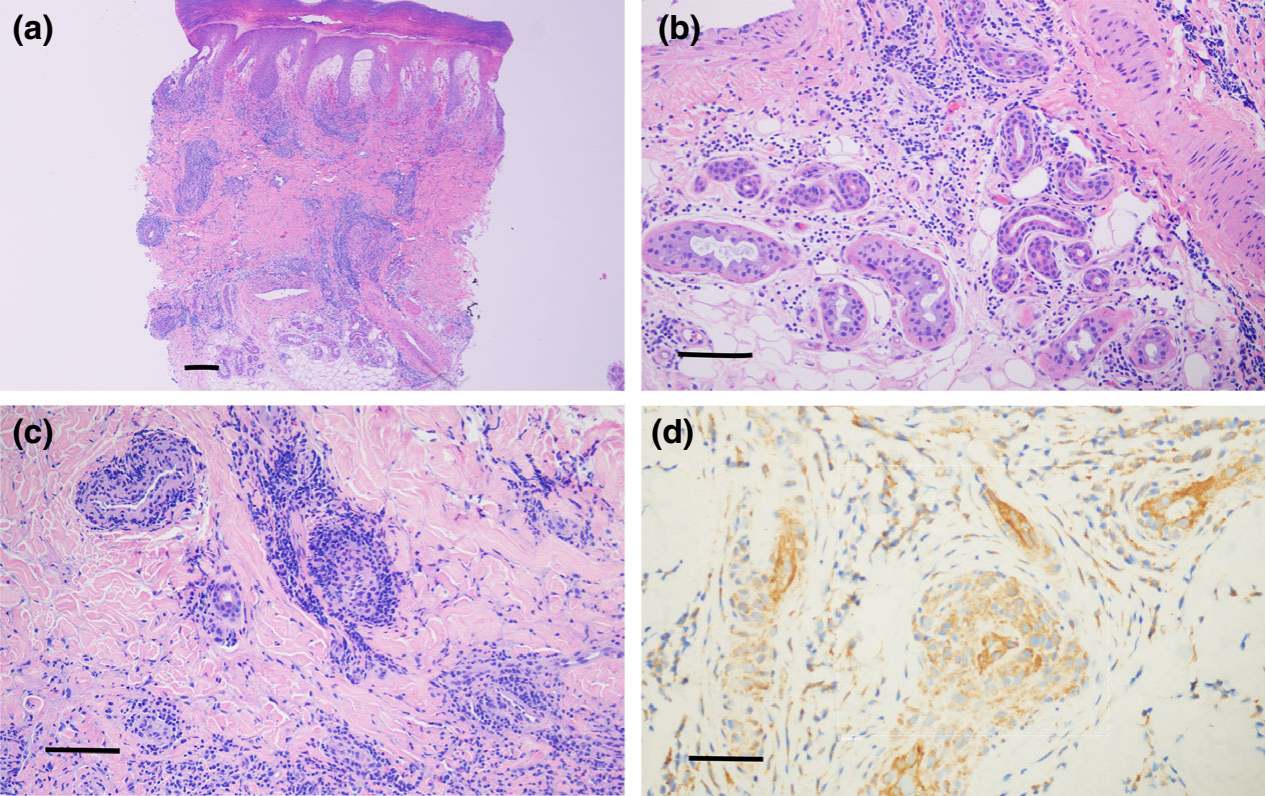
Light microscopic findings and myxovirus resistance protein A (MXA) expression in idiopathic perniosis. (a) In each case there is a superficial and deep angiocentric lymphocytic infiltrate [haematoxylin and eosin (HE), original magnification × 100]. (b) There is a lymphocytic eccrine hidradenitis (HE, × 200). (c) The infiltrates surround and permeate capillaries and venules in the superficial and deep dermis. There are proplastic endothelial cell alterations and focal red cell extravasation without fibrin deposition (HE, × 200). (d) In all cases there is a striking expression of MXA in the epidermis, eccrine ducts and glands, inflammatory cells and endothelium (diaminobenzidine, × 200). Each scale bar is set at 125 microns.

In each case there was a superficial and deep angiocentric lymphocytic infiltrate (Figure 4a). There was lymphocytic eccrine hidradenitis in four of the six cases (Figure 4b). The infiltrate surrounded and permeated capillaries and venules of the superficial and deep dermis (Figure 4c). Vascular fibrin deposition in reticular dermal‐based blood vessels was not seen, although there was endothelial cell swelling and red cell extravasation. Papillary dermal oedema was seen in four of the six cases, with a variable interface dermatitis localized to the tips of the rete ridges (Figure 4a). In all cases there was intense expression of MXA in the epidermis, eccrine ducts and glands, inflammatory cells and endothelium (Figures 2d and 4d).

### COVID‐19‐associated thrombotic retiform purpura

Skin biopsies of an acral fixed livedoid and/or retiform purpuric rash from SARS‐CoV‐2‐positive patients were retrieved from the archival files of the Division of Dermatopathology, Weill Cornell Medicine. The patient cohort was represented by three men who were 70, 71 and 73 years of age and three women who were 36, 40 and 66 years of age (Figure 1c). Two of the patients developed a stroke. Acute renal injury developed in four of the six, requiring dialysis in one. Pre‐existing conditions included hypertension in three, overweight in two, obesity in one, diabetes mellitus in one and hyperlipidaemia in one. The patients had acral livedoid rashes involving the hands bilaterally in three, the plantar aspect of the feet in one and the forearms in two (Figure 1c). All were categorized as being critically ill with COVID‐19 based on prolonged ventilator‐dependent respiratory failure. They had evidence of a hypercoagulable state characterized by elevated D‐dimers ranging from 1026ng mL^−1^ to 40 135ng mL^−1^ (mean 12 027ng mL^−1^) and fibrinogen ranging from 518 to 983 mg dL^−1^ (mean 649 mg dL^−1^), whereby there was a direct parallel between the extent of D‐dimer elevation and fibrinogen elevation. Of the six patients, three died and three had hospitalizations of greater than 2 months.

#### Light microscopic findings and phenotypic studies

**Figure 5:**
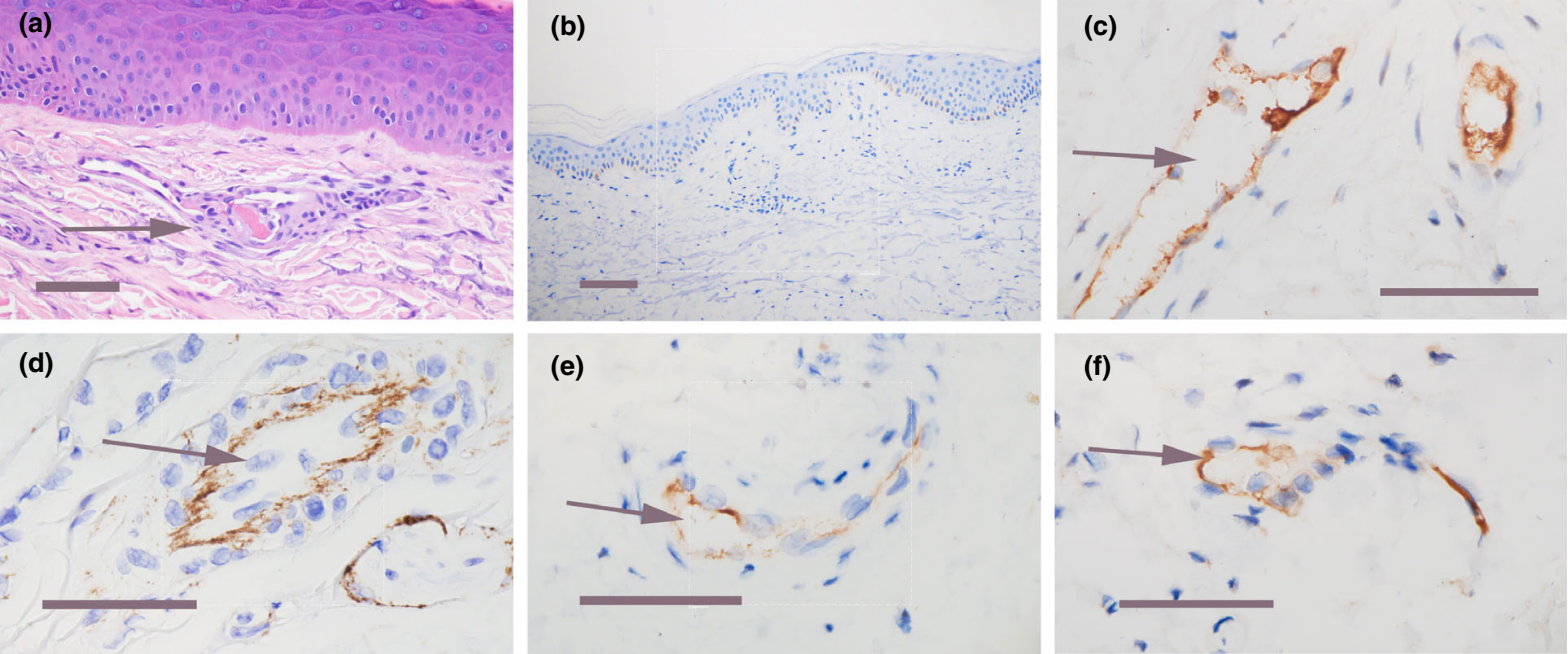
Light microscopic findings, myxovirus resistance protein A (MXA) expression and SARS‐CoV‐2 protein distribution in thrombotic retiform purpura. (a) The biopsies show a pauci‐inflammatory thrombogenic vasculopathy affecting capillaries, venules and arteries within the mid and deeper dermis (haematoxylin and eosin, original magnification × 200). (b) MXA is negative [diaminobenzidine (DAB), × 200]. (c) In all cases of thrombotic retiform purpura, SARS‐CoV‐2 viral spike proteins are readily detected in the endothelia exhibiting an extensive pattern of cytoplasmic immunoreactivity involving both diseased and relatively normal‐appearing blood vessels (DAB, × 1000). (d) Prominent deposits of C5b‐9 are observed within the cutaneous microvasculature of thrombosed and normal‐appearing vessels (DAB, × 1000). (e, f) There is endothelial cell localization of caspase 3 (e, DAB, × 1000) and interleukin‐6 (f, DAB, × 1000) in thrombosed and normal‐appearing blood vessels. Each scale bar is set at 125 microns.

The biopsies showed a pauci‐inflammatory thrombogenic vasculopathy affecting capillaries, venules and arteries (Figure 5a). This was within the mid and deeper dermis and associated with microvascular deposition of C3d, C4d, C5b‐9 and MASP‐2 (Figure 5d). In all cases MXA was negative (Figures 2e and 5b).

#### Viral immunohistochemical protein and RNA assessment in COVID‐19‐associated perniosis and thrombotic retiform purpura of critically ill patients with COVID‐19 and non‐COVID‐19 perniosis

**Figure 6:**
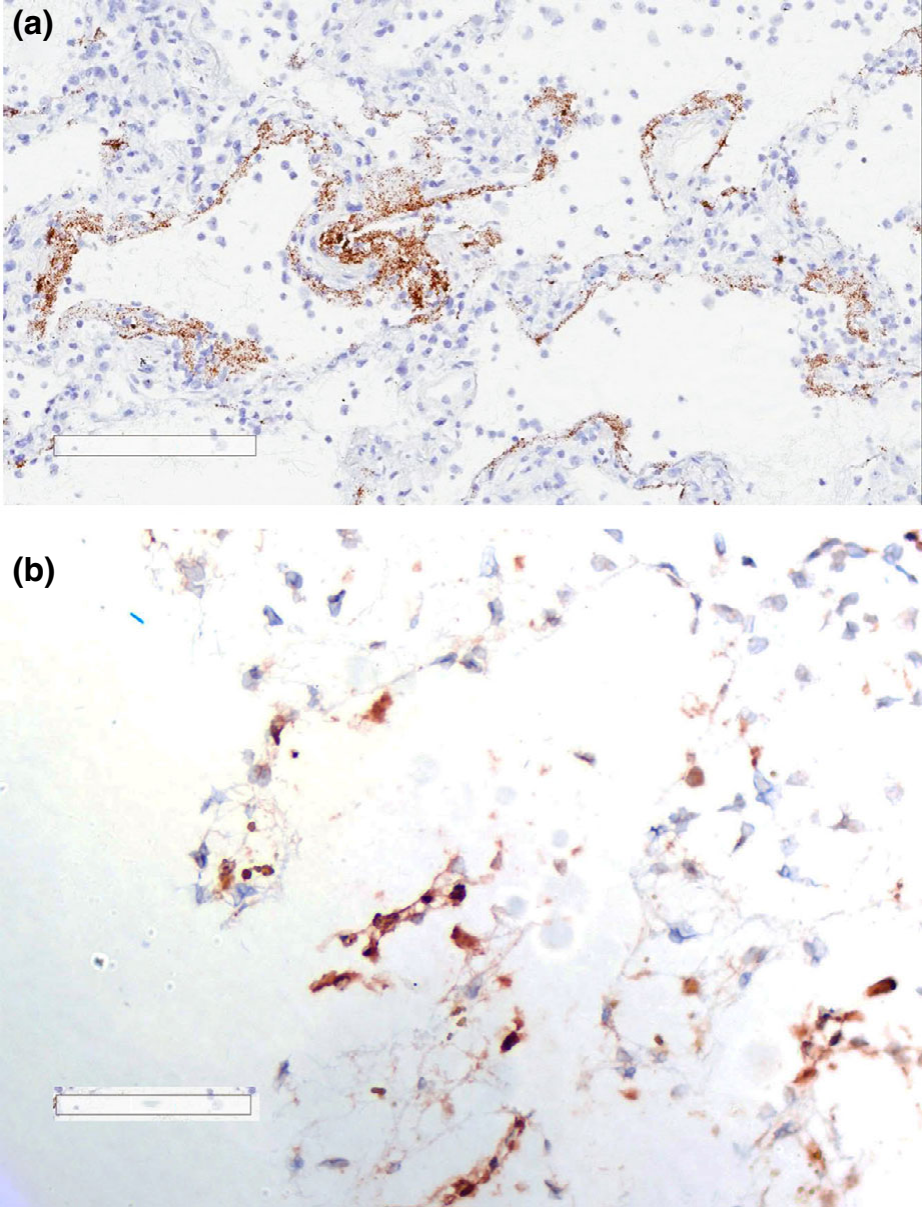
Distribution of SARS‐CoV‐2 viral RNA and SARS‐CoV‐2 protein in a lung sample of a patient with COVID‐19. (a) SARS‐CoV‐2 viral RNA demonstrates striking capillary localization within the interalveolar septa of the lung of a patient who died from COVID‐19 [diaminobenzidine (DAB), original magnification × 200]. (b) SARS‐CoV‐2 membrane protein demonstrated a similar pattern of microvascular localization (DAB, × 400) Scale bars are at 200 microns and 100 microns, respectively.

In the three cases of COVID‐19‐associated perniosis, only rare SARS‐CoV‐2 RNA‐positive cells were present (Figure 3j), while a few mononuclear cells showed detectable SARS‐CoV‐2 membrane and envelope protein (Figure 3k). In all cases of thrombotic retiform purpura, extensive SARS‐CoV‐2 envelope and spike proteins were detected in the endothelial cytoplasms in thrombosed and normal‐appearing blood vessels (Figure 5c), but viral RNA was not detected. The three cases of non‐COVID‐19‐associated perniosis had no detectable protein or virus. For comparison with controls, SARS‐CoV‐2 viral proteins and RNA were examined in the lung of the COVID‐19‐associated fatal pneumonias and normal skin and lung. They were extensively positive in the COVID‐19 lung specimen and negative in the pre‐COVID healthy skin and lung blocks (Figure 6a, b).

#### Interleukin‐6 and caspase 3 studies in COVID‐19‐associated perniosis and thrombotic retiform purpura of critically ill patients with COVID‐19

In the three studied cases of COVID‐19‐associated perniosis only a few cells of probable histiocytic origin expressed interleukin‐6 (Figure 3l). There were scattered inflammatory cells and degenerated keratinocytes positive for caspase 3. Vascular expression was noticeably absent. All cases of thrombotic retiform purpura showed extensive endothelial cell expression of caspase 3 (Figure 5e) and interleukin‐6 (Figure 5f) within the diseased and normal‐appearing blood vessels, mirroring the pattern of SARS‐CoV‐2‐associated protein detection.

## Discussion

We have outlined two disparate cutaneous manifestations of COVID‐19: (i) a highly inflammatory process with minimal vascular injury, manifesting as acral perniosis without significant systemic symptoms and designated as COVID‐19‐associated perniosis, and (ii) a pauci‐inflammatory thrombotic complement‐driven microvascular injury syndrome associated with microangiopathic ARDS.

Chilblains or perniosis represents a robust lymphocyte‐rich vasocentric and perieccrine inflammatory reaction typically found on the digits. Two main variants have been described: (i) cold‐associated idiopathic perniosis, typically seen in young women often with accompanying Raynaud phenomenon, and (ii) secondary perniosis of systemic disease including systemic lupus erythematosus and other autoimmune connective tissue disease (CTD), antiphospholipid antibody syndrome, genetic interferonopathies and viral infections, where cold exposure is an uncommon triggering event.^17^

COVID‐19‐associated chilblain‐like lesions can be considered a form of secondary perniosis. A number of clinical descriptive studies during the COVID‐19 pandemic have reported ‘COVID toes’, reflecting the frequent localization of the eruption to toes, although lesions also occur on the fingers. It is described most frequently in children but has also been reported in adults, including older adults in their eighties and nineties. The cases are typically asymptomatic or mild in character. Key differentiating features clinically from idiopathic perniosis include the absence of any temporal association with cold exposure and no evidence of Raynaud phenomenon. Many cases reported in the literature have not had any COVID‐19 testing, and when COVID‐19 nasopharyngeal swab or serological antibody testing was performed the results were typically negative.^9,10,18–20^ Histological findings encompass a superficial and deep lymphocytic vascular reaction and lymphocytic eccrine hidradenitis similar to conventional perniosis.^9,10,18–20^

The biopsies of our cases of COVID‐19‐associated perniosis showed commonalities with idiopathic perniosis, including lymphocytic infiltration around vessels throughout the dermis and lymphocytic eccrine hidradenitits.^17^ Key discriminating features include pronounced histiocytic infiltration, which included myeloperoxidase‐activated histiocytes and CD11c, and CD123‐positive monocyte‐derived dendritic cells and some degree of frank vasculitic change that could be seen in reticular dermal blood vessels.

The livedoid and retiform skin lesions thus far examined in severe COVID‐19 exhibit a pattern different from those seen in COVID‐19‐associated perniosis. The biopsies in severe cases were pauci‐inflammatory and included features of occlusive fibrin thrombi and striking microvascular complement deposition. The distinct morphologies suggested two different pathophysiological patterns: the mild COVID‐19 disease that prevails in children and the severe or critically ill COVID‐19 disease that occurs in older adults with certain risk factors like obesity and diabetes mellitus.

Idiopathic/familial perniosis, chilblain lupus and Aicardi–Goutières syndrome are associated with mutations in *TREX1* and *RNASEH2A*. Aicardi–Goutières syndrome is characterized by perniosis, periodic fevers and microcephaly, with progressive intellectual impairment due to cerebral lymphocytic vasculitis. The end result of these mutations is excessive interferon signalling. It has been suggested that these nucleases are involved in removing nucleic acids that accumulate during apoptosis, and when this process fails, an interferon signal is generated that activates the immune system. MXA, the surrogate marker for type I interferon, would be expected to be significantly upregulated in idiopathic/familial perniosis, chilblain lupus and perniosis in the setting of Aicardi–Goutières syndrome.^21^

In this current study, MXA was uniformly expressed at high levels in our cases of idiopathic/familial perniosis, as revealed by striking MXA expression in the epidermis, endothelium and inflammatory cells. The cases of COVID‐19‐associated perniosis showed extensive expression in the epidermis, endothelium and inflammatory cells for MXA, similar to idiopathic perniosis and secondary perniosis in the setting of excessive interferon signalling (i.e. chilblain lupus and Aicardi–Goutières syndrome). The nature of the infiltrate in this small study cohort is reflective of the type I interferon‐enriched microenvironment, being one rich in CD14^+^ CD11c^+^ monocyte‐derived dendritic cells including many with a classic CD4^+^ CD123^+^ plasmacytoid dendritic cell phenotype, activated myeloperoxidase‐positive macrophages, and T cells with only rare B cells.^22^–^24^

The negative nasopharyngeal swabs in our two patients with COVID‐19 perniosis may reflect the efficacy of interferon‐driven monocyte and T‐cell responses to clear the virus in these patients, and appear to be an almost ubiquitous finding in patients with COVID‐19‐associated perniosis.^9,10,17–19^ Furthermore, serological testing was negative in one of our patients despite demonstration of SARS‐CoV‐2 viral proteins and RNA in the patient’s skin lesions. More studies are needed to determine why patients with COVID‐19‐associated perniosis could have negative antibody testing, but the timing and sensitivity of the test and the individual immune responses to the virus are all likely contributors. A similar type I interferon‐induced T helper 1 and monocyte response is seen in classic Kawasaki disease and accounts for the T‐cell and monocyte‐rich arteritis seen in these patients. By extension it is possible that the recently described multiorgan Kawasaki‐like illness rarely seen in children with COVID‐19 infection could develop as a result of excessively high levels of type I interferon.^11^, ^25^, ^26^

In contradistinction, the absent MXA expression in the skin biopsies from severe or critically ill patients with COVID‐19 suggests a lack of a type I interferon response. Certain disease states can suppress type I interferon response, including obesity, which is associated with worse COVID‐19 outcomes.^27^ The production of the adipokine leptin is proportional to the body mass index, and its production is regulated by suppressor of cytokine signalling (SOCS)3. Terán‐Cabanillas and Hernández showed that when SOCS3 is upregulated in a high‐leptin state, in addition to leptin it suppresses the type I interferon response.^28^ Type I interferons, unlike the more discussed type II interferons (i.e. interferon‐γ), can be induced in all cell types, but are silent in a state of health. In the case of COVID‐19, type I interferons are induced by single‐stranded RNA of the virus binding to Toll‐like receptors 7 and 8 in endosomes. The type I interferons produced are released and bind to the type I interferon homodimer receptor, which is ubiquitous in the body.^29^ This leads to signalling through signal transducer and activator of transcription (STAT)1 and STAT2, which starts the production of a host of protective proteins, including interferon‐induced transmembrane protein (IFITM)3, which acts as a complement regulator.^30^ A polymorphism in IFITM3 has been shown to lead to poor outcomes in COVID‐19 and is a topic that warrants further study.^31^

We propose that a strong type I interferon response may speed viral elimination, whereas a blunted type I interferon response would allow for massive viral replication leading to complement activation through the mannan‐binding lectin pathway. It has been noted that interferon I response is often impaired in critically ill patients,^31^ and that association plays out in transcriptional analysis of interferon in SARS‐CoV‐2‐infected, angiotensin‐converting enzyme 2‐expressing cells *in vitro*.^32^ Not surprisingly, SARS‐CoV‐2 is susceptible to type I interferon pretreatment,^32^ as shown in a currently unpublished study from Vineet Menachery, lending further credence to the benefits of a strong type I interferon response. This hypothesis is shared by a group at Yale, who wrote an editorial that reviewed some of the existing literature on COVID‐19‐associated perniosis and suggested its potential pathophysiological commonality with idiopathic perniosis as a type of interferonopathy, although specific data supporting that hypothesis were not presented.^33^

The amount and microanatomical distribution of viral protein expression in COVID‐19‐associated perniosis, in contrast to the thrombotic purpuric lesions of severe or critically ill COVID‐19, reflects the importance of the antiviral effects of type I interferons. In the cases of perniosis studied, there was no expression of SARS‐CoV‐2 protein in endothelium, and only a few cells of probable monocytic lineage contained SARS‐CoV‐2 protein and RNA. In the retiform thrombotic lesions, there was extensive localization of SARS‐CoV‐2 protein in the endothelium, although without any evidence of viral replication. There is evidence that the SARS‐CoV‐2 spike protein is able to engage the mannose‐binding lectin (MBL) pathway, resulting in MASP‐2 activation and subsequent formation of the C3 convertase and ultimately C5b‐9 (i.e. the membranolytic attack complex). This is the effector mechanism of endothelial cell injury and subsequent thrombosis that we and others have previously reported.^5^, ^33^, ^34^ Furthermore, MBL activation leads to further systemic amplification of the alternative pathway and activation of the coagulation pathway, along with well‐established feedback‐loop mechanisms. The striking expression of the proapoptotic marker caspase 3 likely reflects the sequelae of MBL activation, which is known to result in apoptosis via caspase 3 activation.^35^

The extensive degree of interleukin‐6 upregulation in endothelium could be due to its elaboration by endothelial cells via MBL activation, as suggested in two separate studies.^36^, ^37^ Interleukin‐6 may be involved in the pathogenesis of the vascular thrombosis through its effects on platelet aggregation and activation,^38^, ^39^ or upon angiotensin II regulation,^40^, ^41^ or it may simply be a bystander resulting from hypoxia.

In conclusion, patients with COVID‐19‐associated perniosis demonstrate a robust immune reaction characterized by a T‐cell and interferon‐rich microenvironment, which we believe leads to a mild course of the viral illness. The extent of interferon‐driven inflammation that can occur in children is clearly effective in eliminating the virus, but could also have deleterious consequences if unleashed in a multiorgan inflammatory context. In the pauci‐inflammatory thrombosed skin of very ill patients with COVID‐19, interferon signalling is essentially absent, the implication being a permissive microenvironment that allows unchecked viral replication and triggers extensive complement activation of both the alternative pathway and the MBL pathway with catastrophic sequelae.

## Acknowledgments

We thank Dr Ty Hanson from Sioux Falls, South Dakota for providing case material. We also thank Bing He from Weill Cornell Medicine, Department of Pathology and Laboratory Medicine and the TRP Research lab for their superb technical assistance and advice.

## Supplementary Material

bjd19415-sup-0001-JournalClub **Powerpoint S1** Journal Club Slide Set.

bjd19415-sup-0002-VideoS1 **Video S1** Author video.
