# Supplementary material for: The differing pathophysiologies that underlie COVID‐19‐associated perniosis and thrombotic retiform purpura: a case series
Source: Br J Dermatol. 2021 Jan 1;184(1):141–50. doi: 10.1111/bjd.19415 (PMC7405151; doi:10.1111/bjd.19415)
Supplement: bjd19415-sup-0001-JournalClub — Powerpoint S1 Journal Club Slide Set. [file bjd19415-sup-0001-journalclub.pptx]

## Slide 1
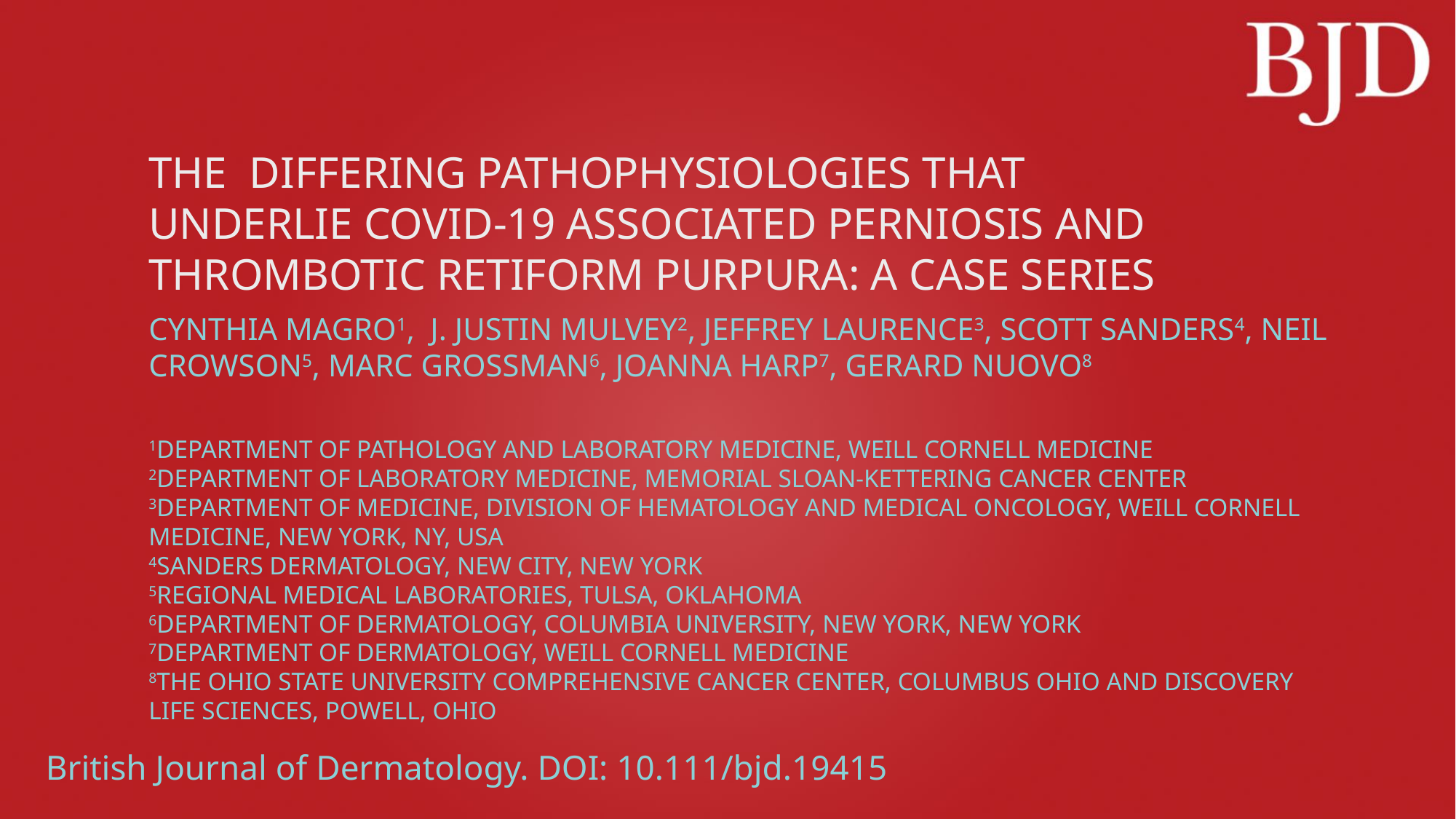

# THE DIFFERING PATHOPHYSIOLOGIES THAT UNDERLIE COVID-19 ASSOCIATED PERNIOSIS AND THROMBOTIC RETIFORM PURPURA: A CASE SERIES
Cynthia Magro1, J. Justin Mulvey2, Jeffrey Laurence3, Scott Sanders4, Neil Crowson5, Marc Grossman6, Joanna Harp7, Gerard Nuovo8
1Department of Pathology and Laboratory Medicine, Weill Cornell Medicine
2Department of Laboratory Medicine, Memorial Sloan-Kettering Cancer Center
3Department of Medicine, Division of Hematology and Medical Oncology, Weill Cornell Medicine, New York, NY, USA
4Sanders Dermatology, New City, New York
5Regional Medical Laboratories, Tulsa, Oklahoma
6Department of Dermatology, Columbia University, New York, New York
7Department of Dermatology, Weill Cornell Medicine
8The Ohio State University Comprehensive Cancer Center, Columbus Ohio and Discovery Life Sciences, Powell, Ohio
British Journal of Dermatology. DOI: 10.111/bjd.19415

## Slide 2
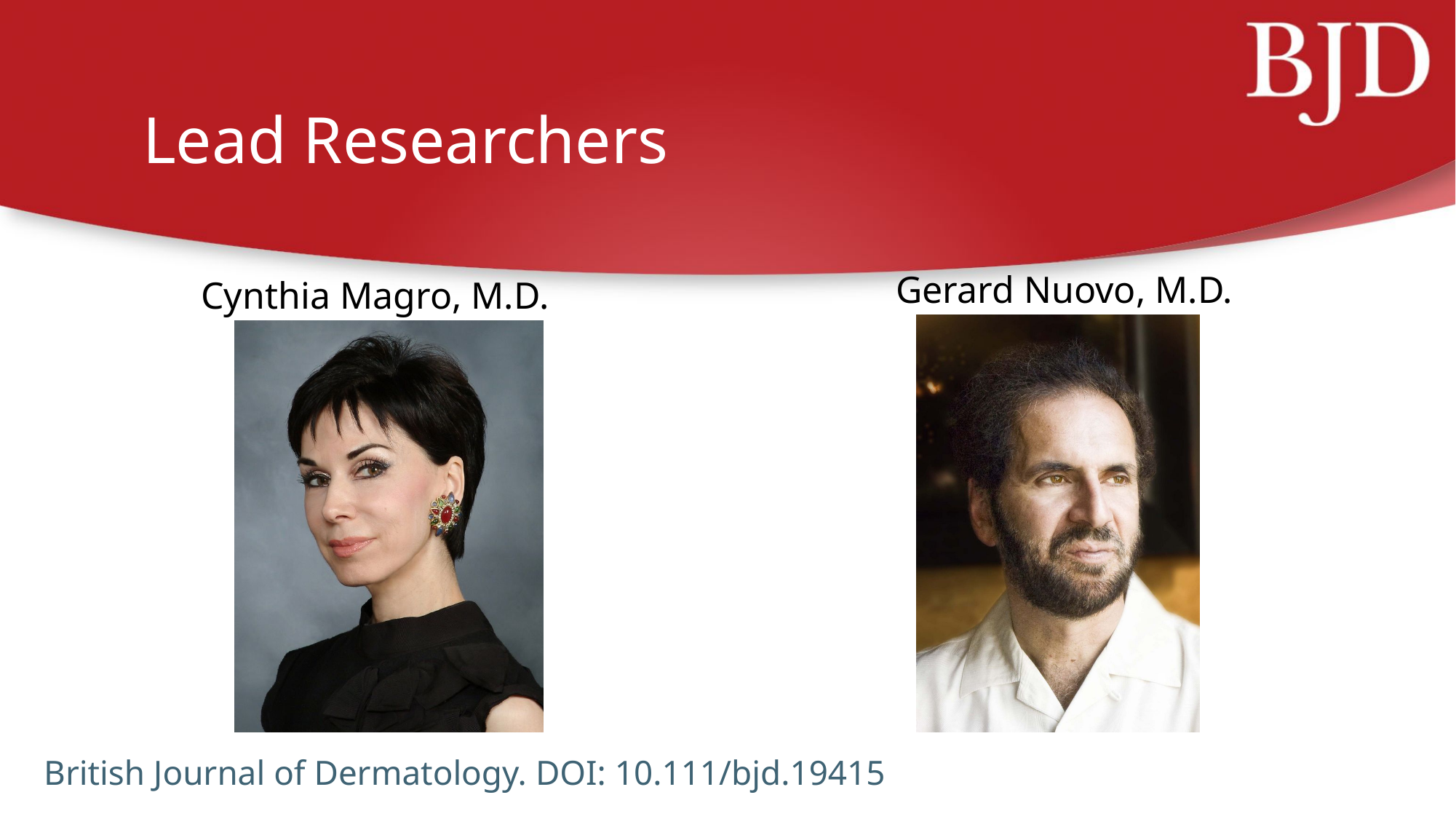

# Lead Researchers
Gerard Nuovo, M.D.
Cynthia Magro, M.D.
British Journal of Dermatology. DOI: 10.111/bjd.19415

## Slide 3
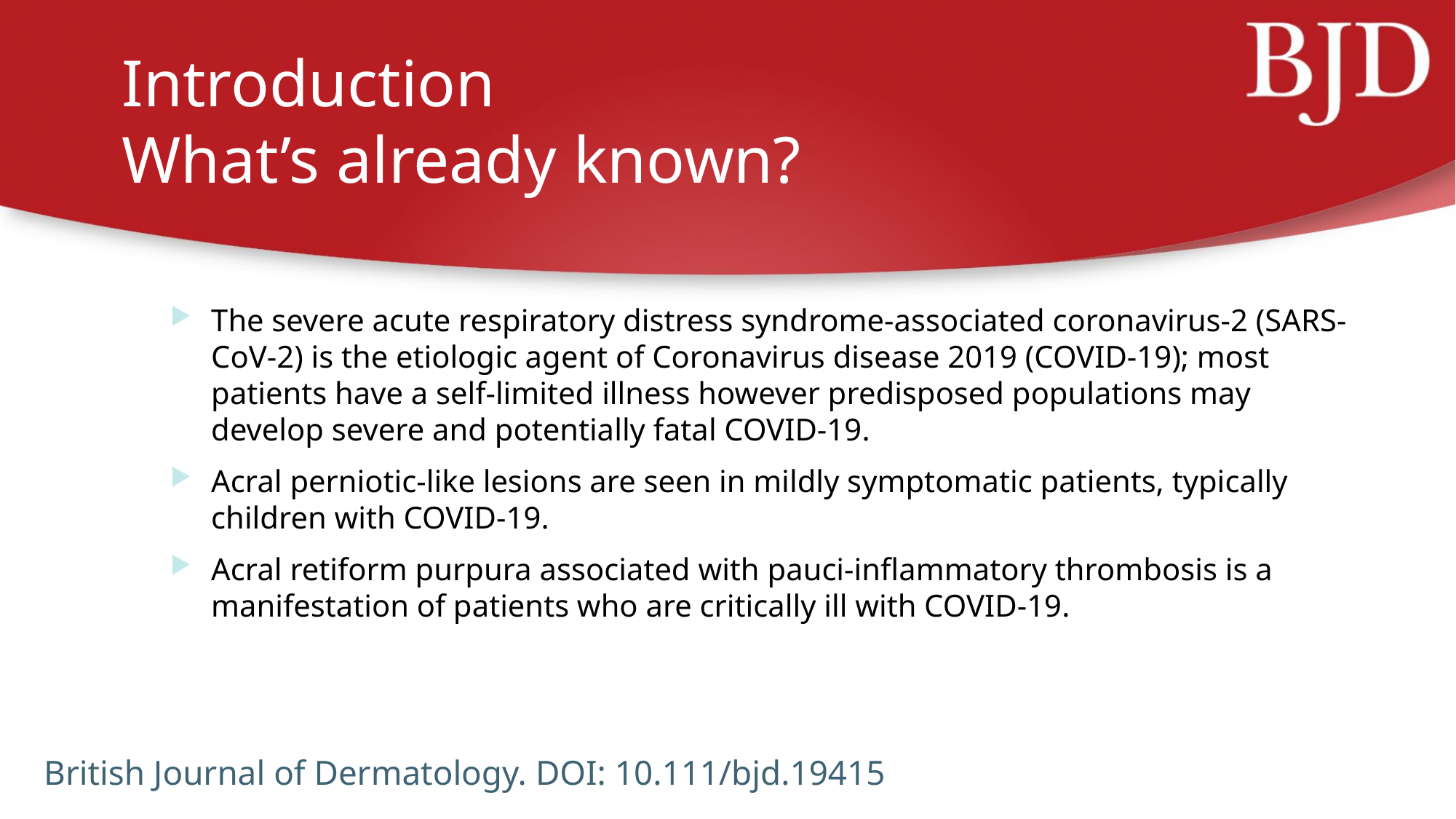

# Introduction What’s already known?
The severe acute respiratory distress syndrome-associated coronavirus-2 (SARS-CoV-2) is the etiologic agent of Coronavirus disease 2019 (COVID-19); most patients have a self-limited illness however predisposed populations may develop severe and potentially fatal COVID-19.
Acral perniotic-like lesions are seen in mildly symptomatic patients, typically children with COVID-19.
Acral retiform purpura associated with pauci-inflammatory thrombosis is a manifestation of patients who are critically ill with COVID-19.
British Journal of Dermatology. DOI: 10.111/bjd.19415

## Slide 4
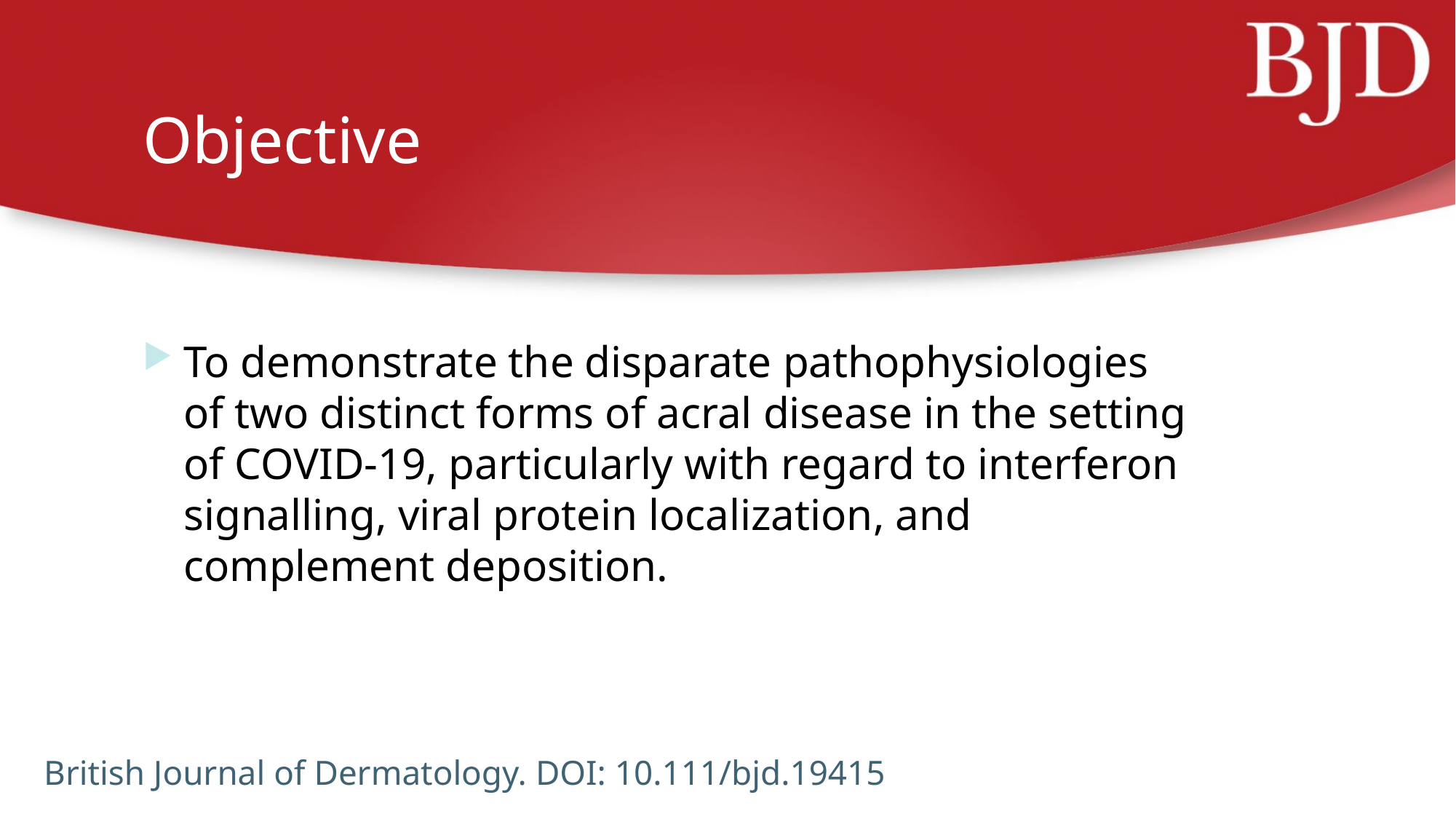

# Objective
To demonstrate the disparate pathophysiologies of two distinct forms of acral disease in the setting of COVID-19, particularly with regard to interferon signalling, viral protein localization, and complement deposition.
British Journal of Dermatology. DOI: 10.111/bjd.19415

## Slide 5
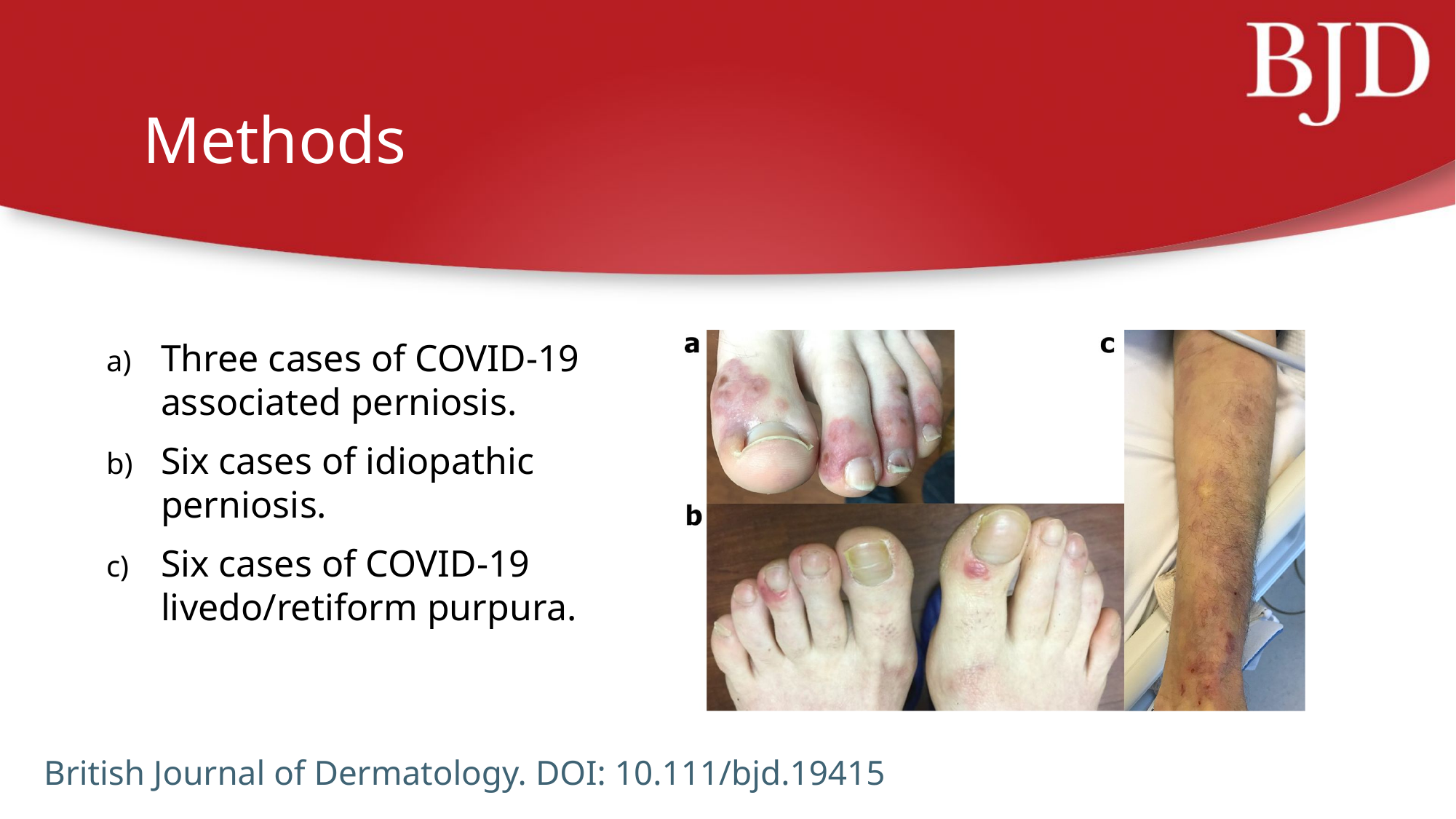

# Methods
Three cases of COVID-19 associated perniosis.
Six cases of idiopathic perniosis.
Six cases of COVID-19 livedo/retiform purpura.
British Journal of Dermatology. DOI: 10.111/bjd.19415

## Slide 6
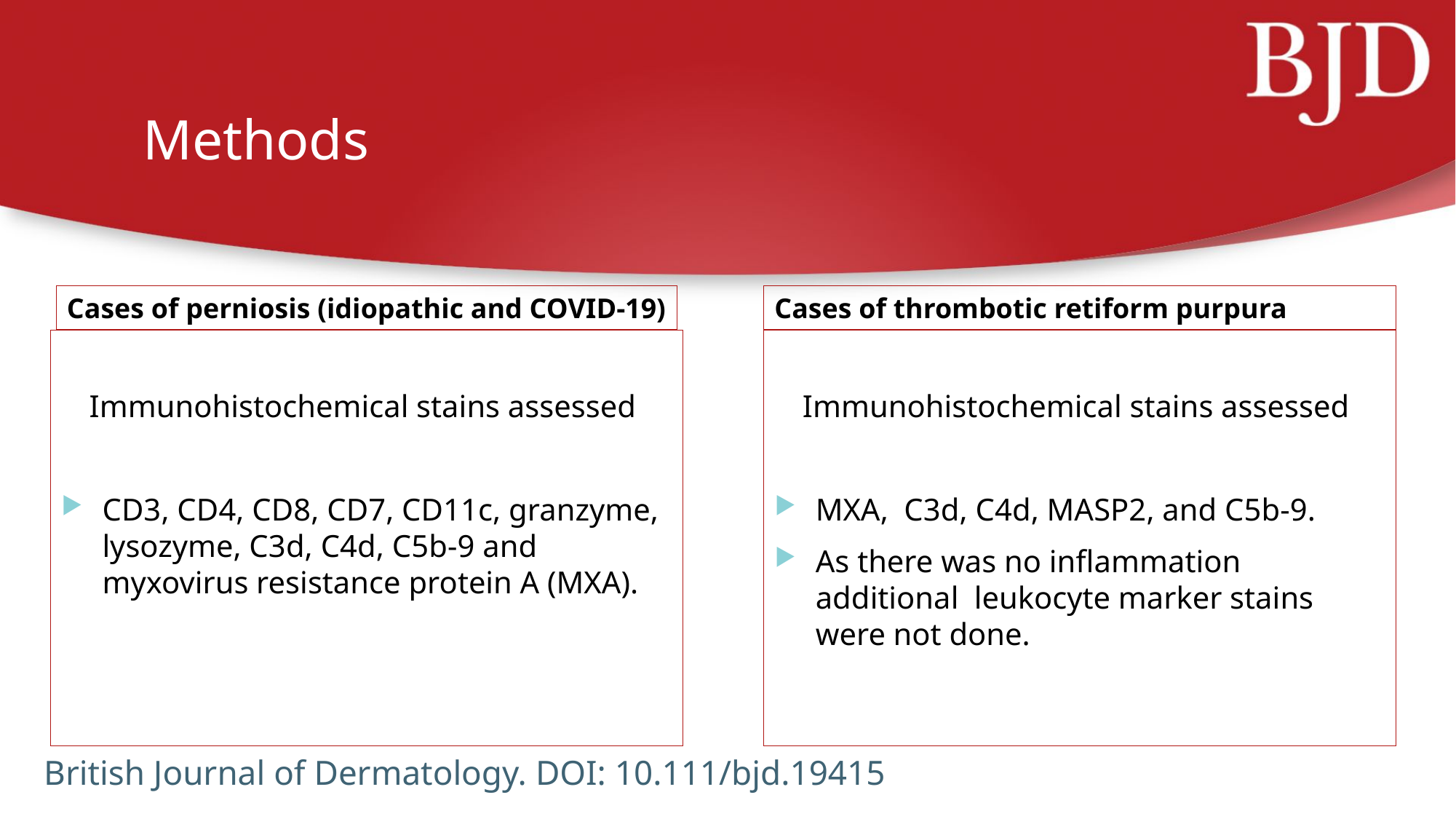

# Methods
Cases of perniosis (idiopathic and COVID-19)
Cases of thrombotic retiform purpura
Immunohistochemical stains assessed
MXA, C3d, C4d, MASP2, and C5b-9.
As there was no inflammation additional leukocyte marker stains were not done.
Immunohistochemical stains assessed
CD3, CD4, CD8, CD7, CD11c, granzyme, lysozyme, C3d, C4d, C5b-9 and myxovirus resistance protein A (MXA).
British Journal of Dermatology. DOI: 10.111/bjd.19415

## Slide 7
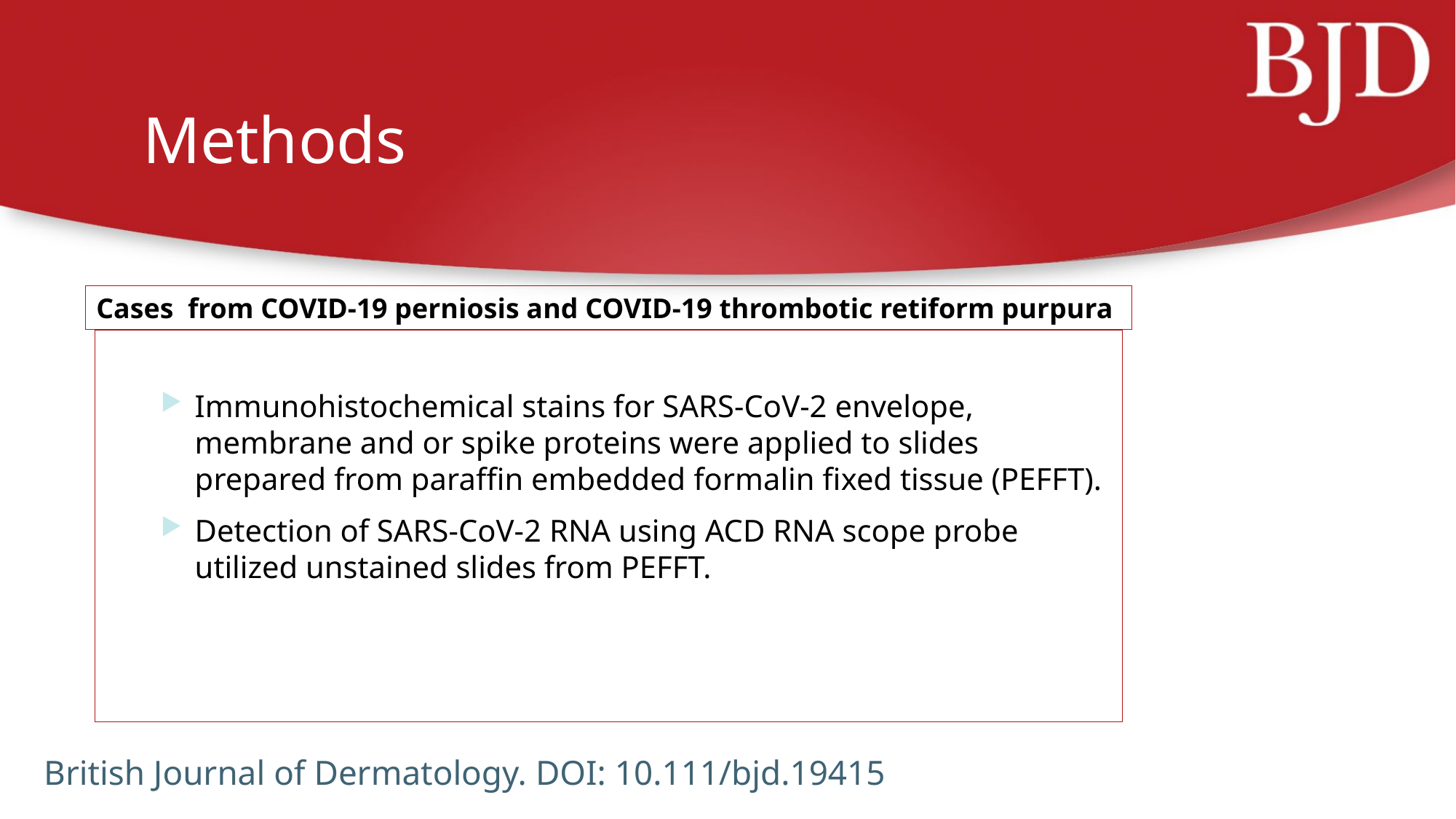

# Methods
Cases from COVID-19 perniosis and COVID-19 thrombotic retiform purpura
Immunohistochemical stains for SARS-CoV-2 envelope, membrane and or spike proteins were applied to slides prepared from paraffin embedded formalin fixed tissue (PEFFT).
Detection of SARS-CoV-2 RNA using ACD RNA scope probe utilized unstained slides from PEFFT.
British Journal of Dermatology. DOI: 10.111/bjd.19415

## Slide 8
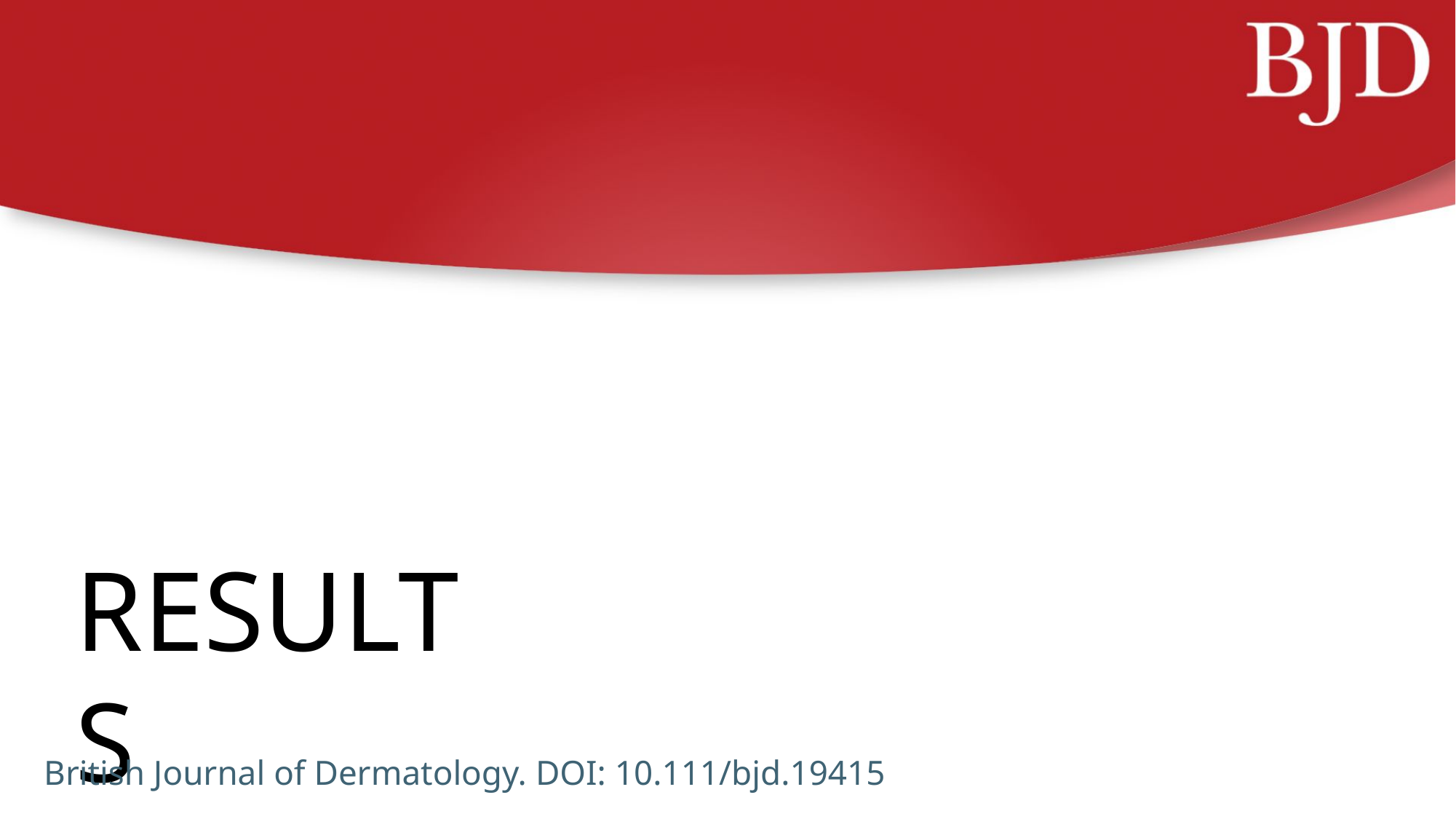

RESULTS
British Journal of Dermatology. DOI: 10.111/bjd.19415

## Slide 9
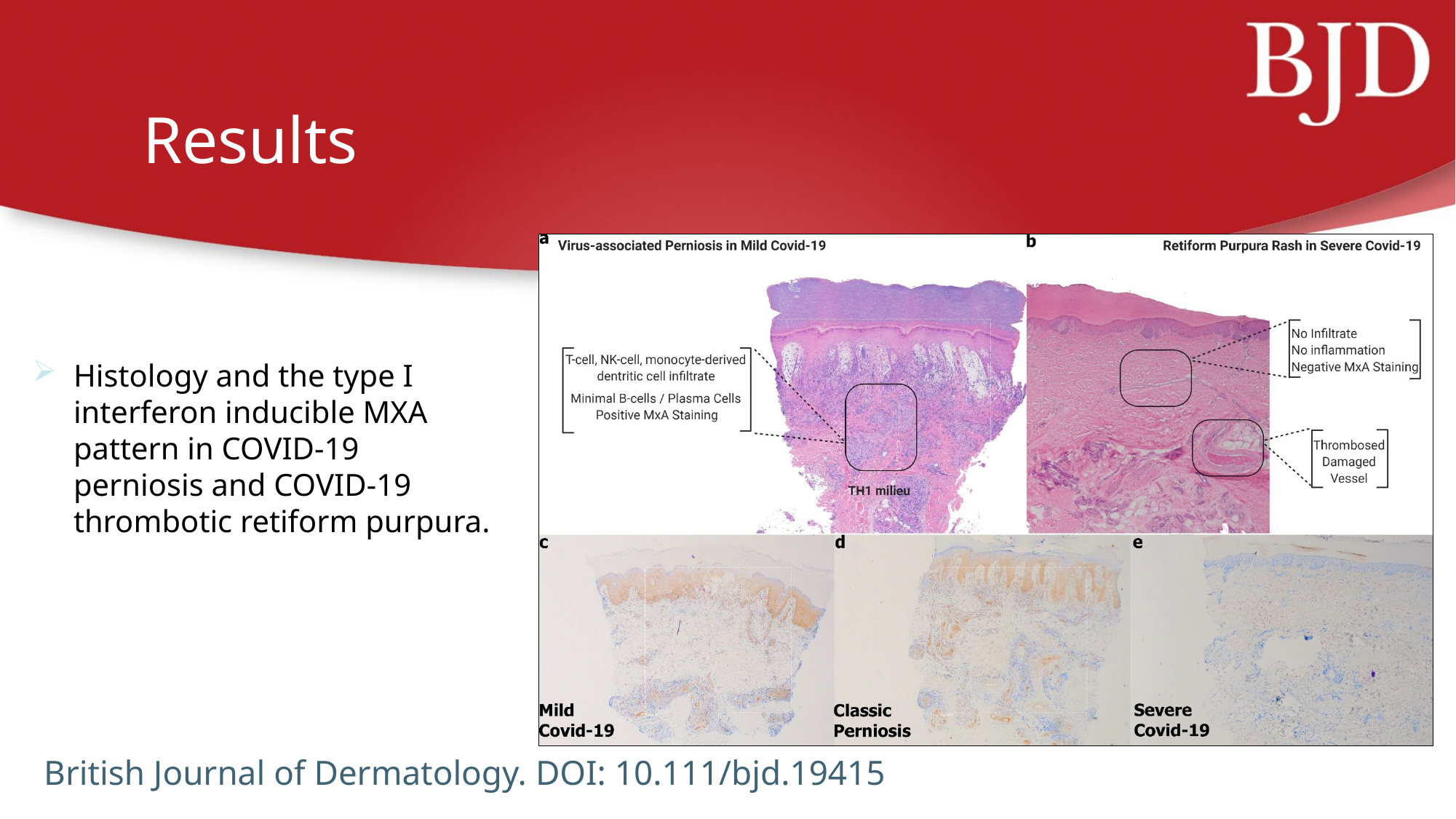

# Results
Histology and the type I interferon inducible MXA pattern in COVID-19 perniosis and COVID-19 thrombotic retiform purpura.
British Journal of Dermatology. DOI: 10.111/bjd.19415

## Slide 10
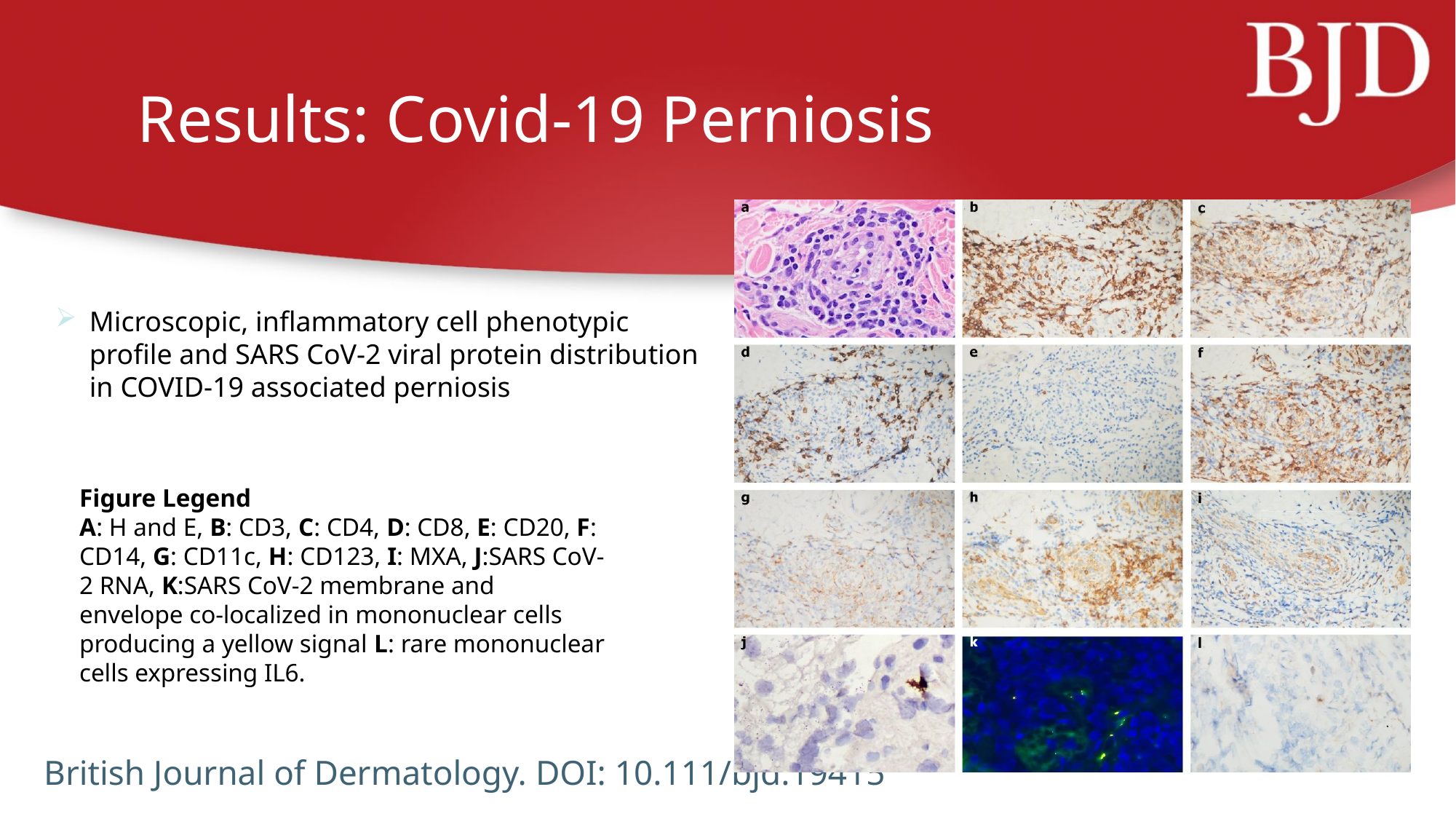

# Results: Covid-19 Perniosis
Microscopic, inflammatory cell phenotypic profile and SARS CoV-2 viral protein distribution in COVID-19 associated perniosis
Figure Legend
A: H and E, B: CD3, C: CD4, D: CD8, E: CD20, F: CD14, G: CD11c, H: CD123, I: MXA, J:SARS CoV-2 RNA, K:SARS CoV-2 membrane and envelope co-localized in mononuclear cells producing a yellow signal L: rare mononuclear cells expressing IL6.
British Journal of Dermatology. DOI: 10.111/bjd.19415

## Slide 11
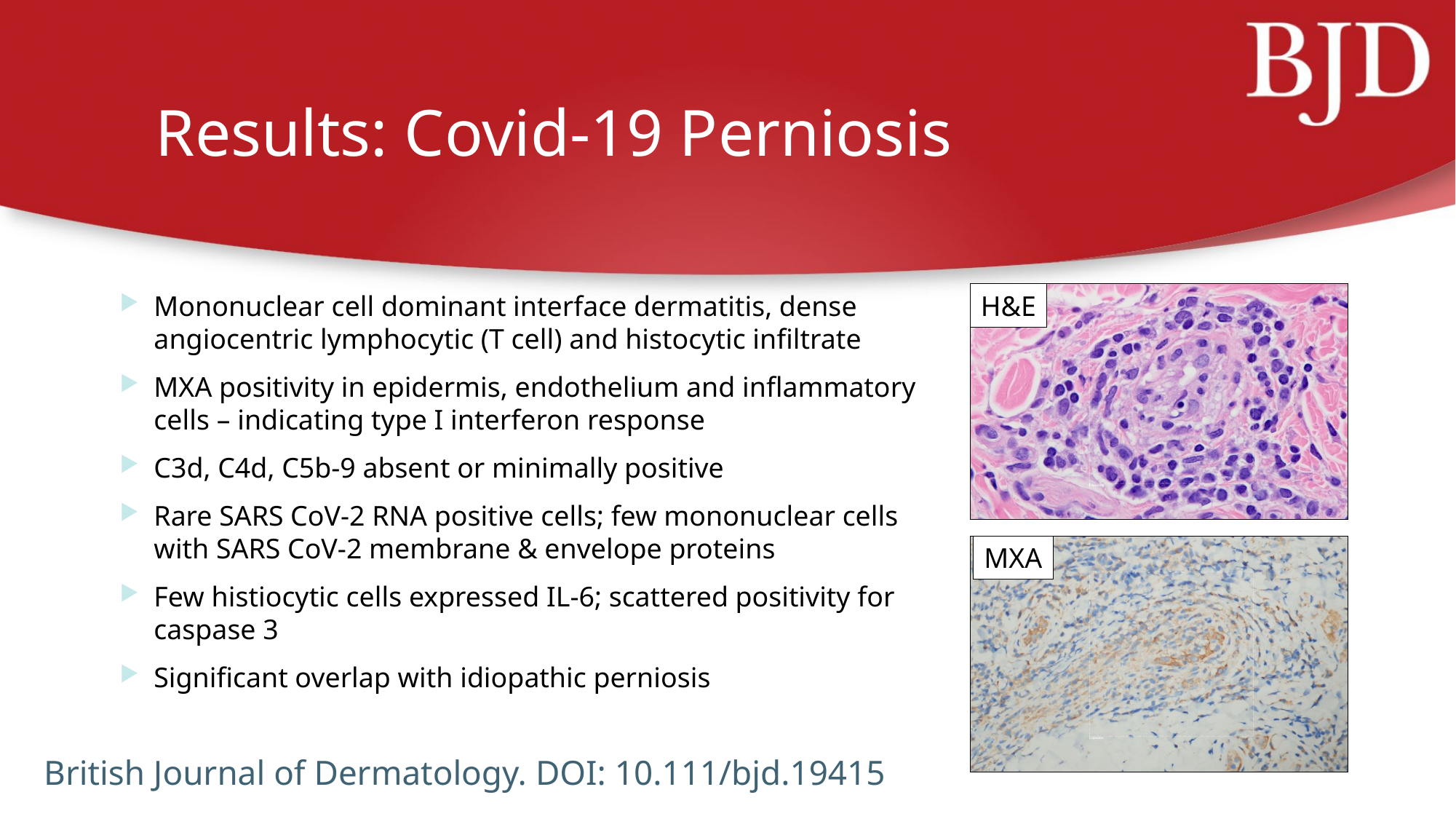

Results: Covid-19 Perniosis
Mononuclear cell dominant interface dermatitis, dense angiocentric lymphocytic (T cell) and histocytic infiltrate
MXA positivity in epidermis, endothelium and inflammatory cells – indicating type I interferon response
C3d, C4d, C5b-9 absent or minimally positive
Rare SARS CoV-2 RNA positive cells; few mononuclear cells with SARS CoV-2 membrane & envelope proteins
Few histiocytic cells expressed IL-6; scattered positivity for caspase 3
Significant overlap with idiopathic perniosis
H&E
MXA
British Journal of Dermatology. DOI: 10.111/bjd.19415

## Slide 12
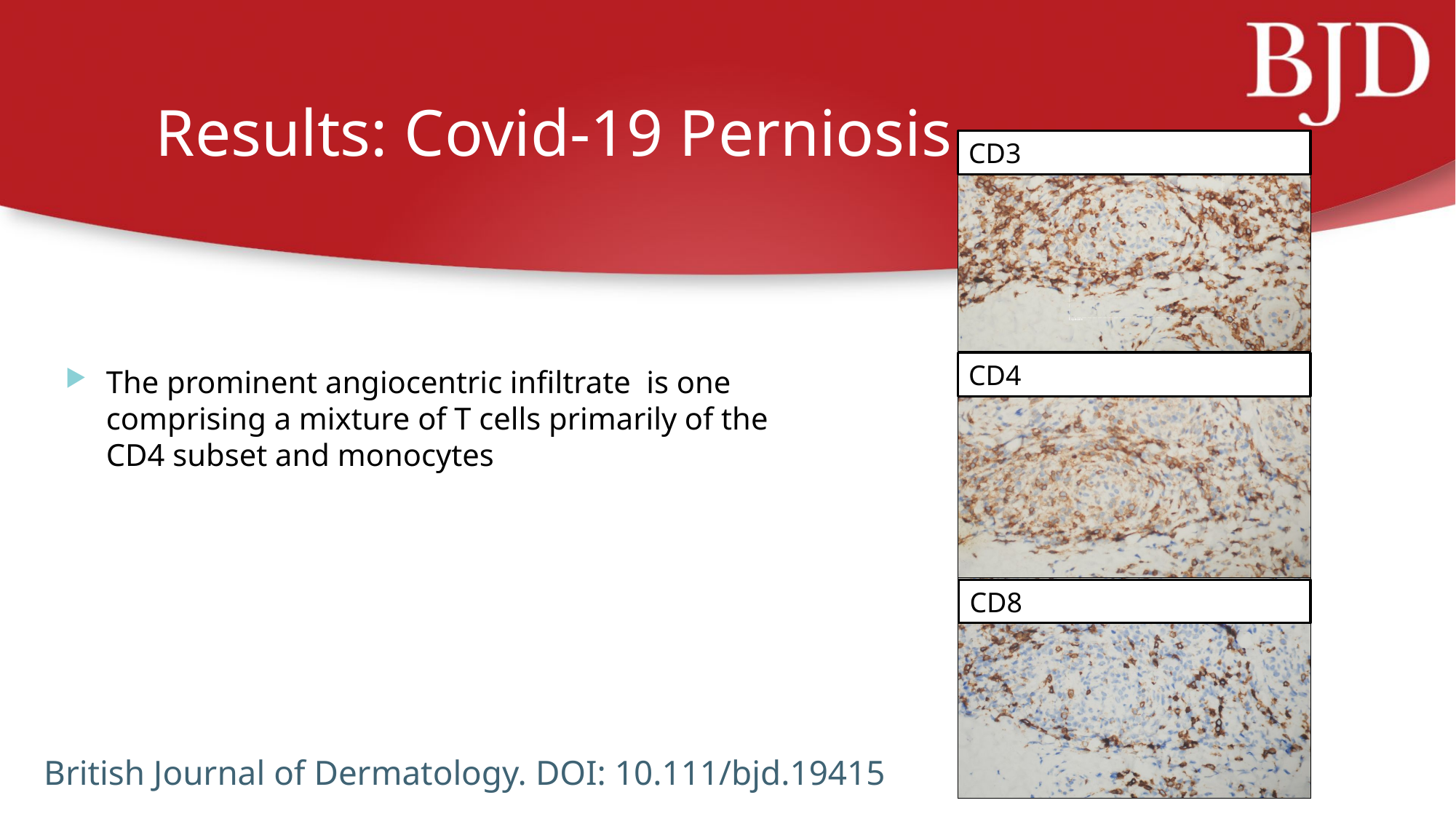

Results: Covid-19 Perniosis
CD3
CD4
The prominent angiocentric infiltrate is one comprising a mixture of T cells primarily of the CD4 subset and monocytes
CD8
British Journal of Dermatology. DOI: 10.111/bjd.19415

## Slide 13
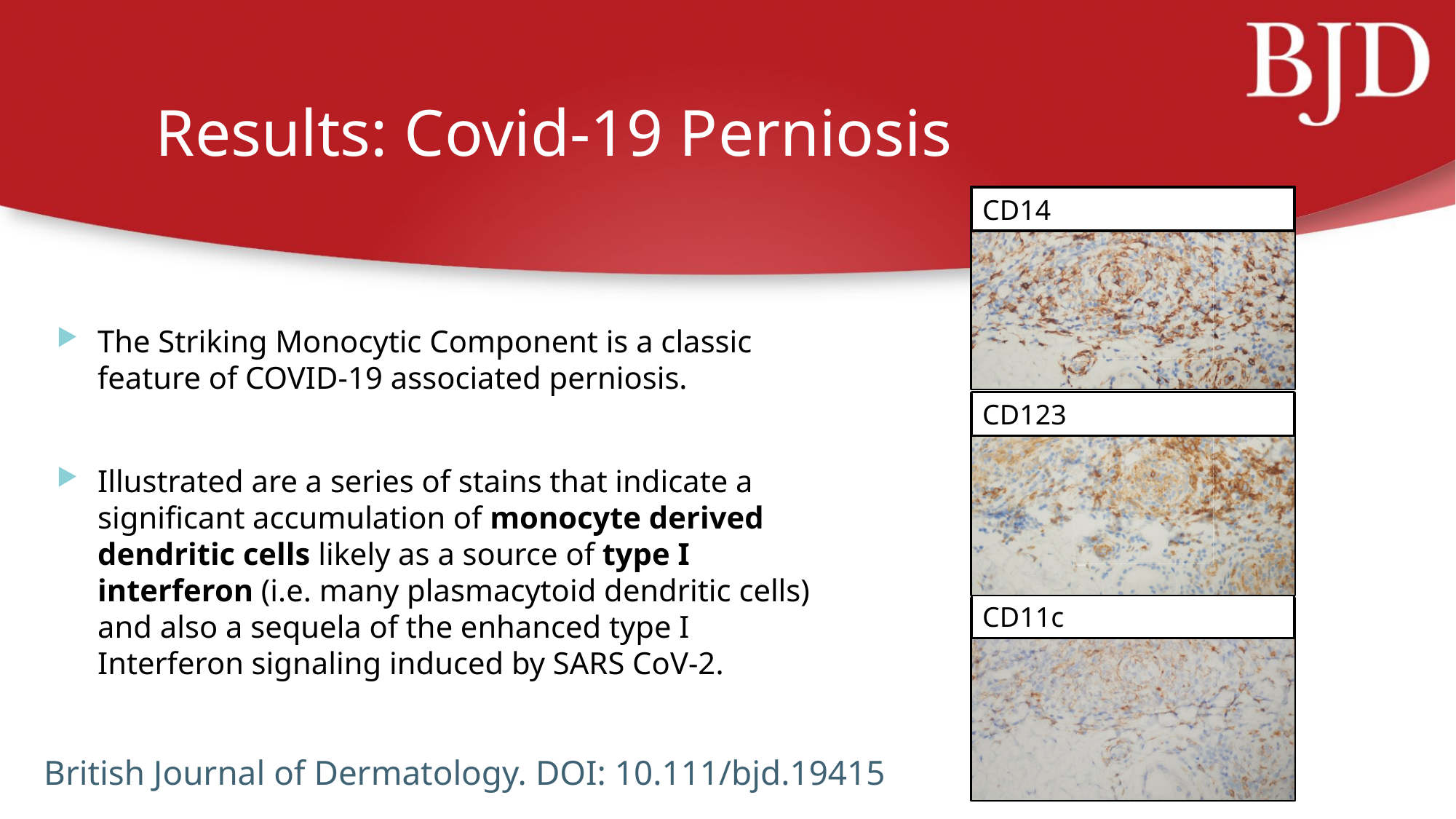

Results: Covid-19 Perniosis
CD14
The Striking Monocytic Component is a classic feature of COVID-19 associated perniosis.
Illustrated are a series of stains that indicate a significant accumulation of monocyte derived dendritic cells likely as a source of type I interferon (i.e. many plasmacytoid dendritic cells) and also a sequela of the enhanced type I Interferon signaling induced by SARS CoV-2.
CD123
CD11c
British Journal of Dermatology. DOI: 10.111/bjd.19415

## Slide 14
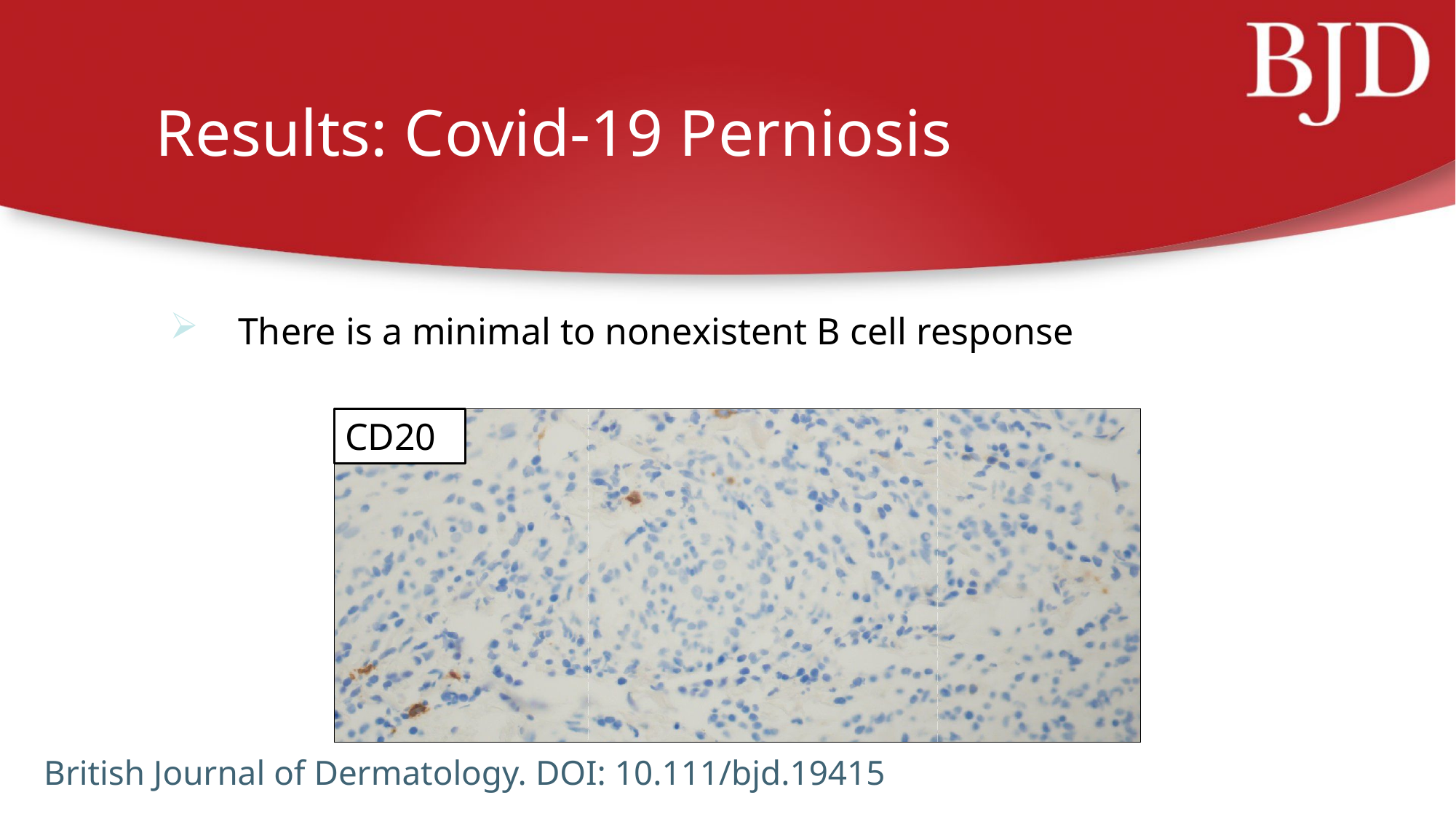

Results: Covid-19 Perniosis
# There is a minimal to nonexistent B cell response
CD20
British Journal of Dermatology. DOI: 10.111/bjd.19415

## Slide 15
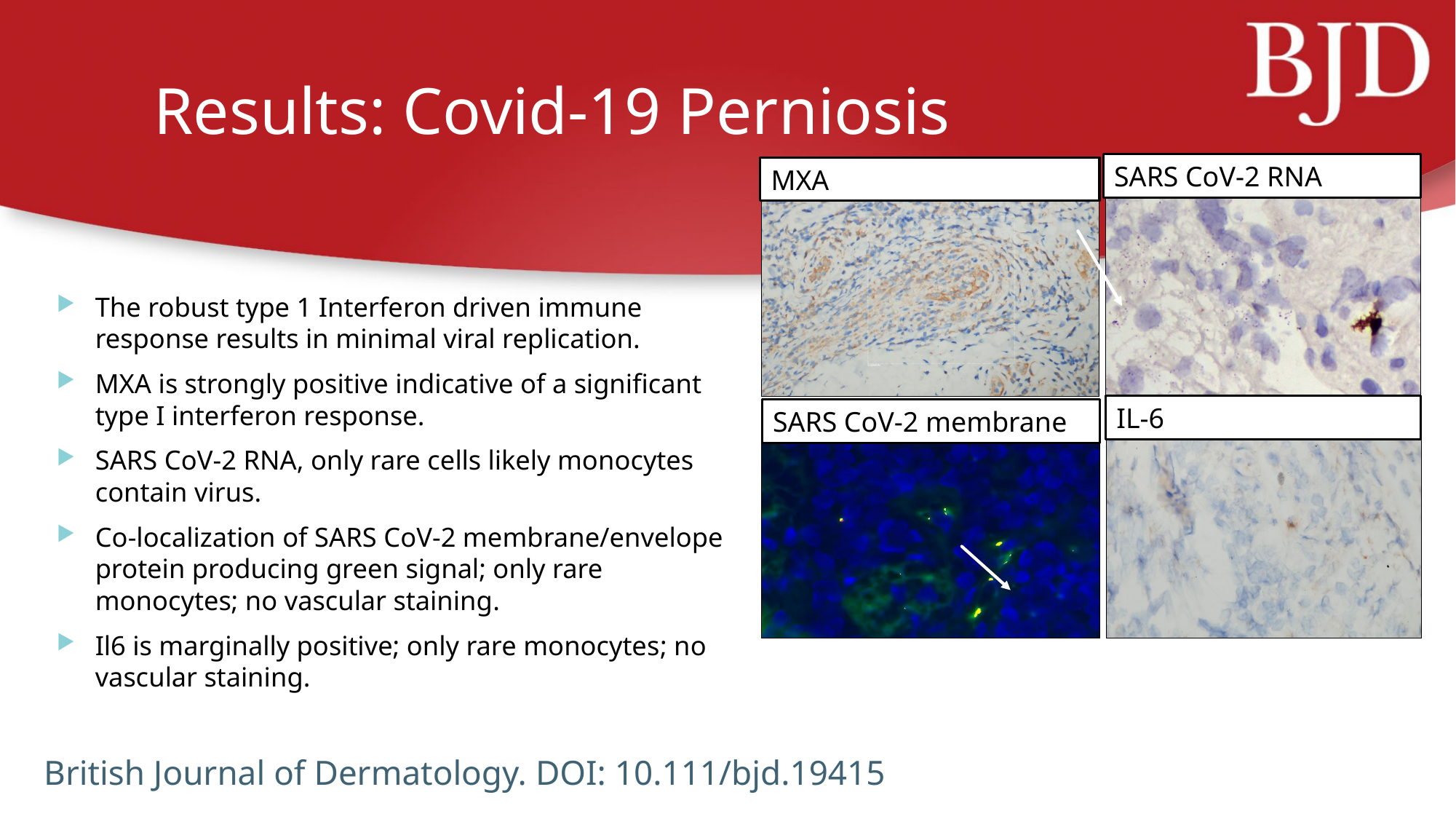

Results: Covid-19 Perniosis
SARS CoV-2 RNA
MXA
The robust type 1 Interferon driven immune response results in minimal viral replication.
MXA is strongly positive indicative of a significant type I interferon response.
SARS CoV-2 RNA, only rare cells likely monocytes contain virus.
Co-localization of SARS CoV-2 membrane/envelope protein producing green signal; only rare monocytes; no vascular staining.
Il6 is marginally positive; only rare monocytes; no vascular staining.
IL-6
SARS CoV-2 membrane
British Journal of Dermatology. DOI: 10.111/bjd.19415

## Slide 16
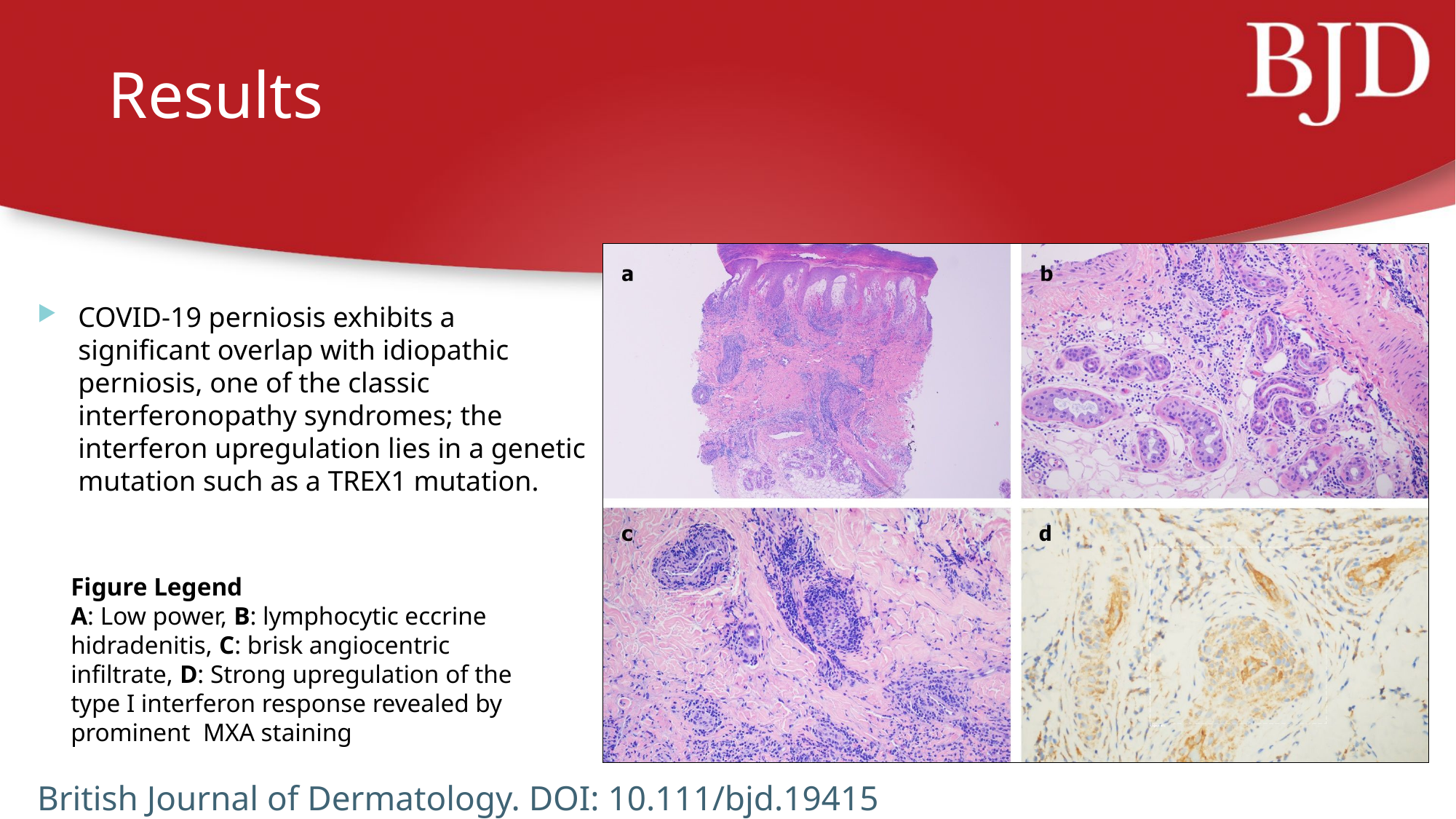

Results
COVID-19 perniosis exhibits a significant overlap with idiopathic perniosis, one of the classic interferonopathy syndromes; the interferon upregulation lies in a genetic mutation such as a TREX1 mutation.
Figure Legend
A: Low power, B: lymphocytic eccrine hidradenitis, C: brisk angiocentric infiltrate, D: Strong upregulation of the type I interferon response revealed by prominent MXA staining
British Journal of Dermatology. DOI: 10.111/bjd.19415

## Slide 17
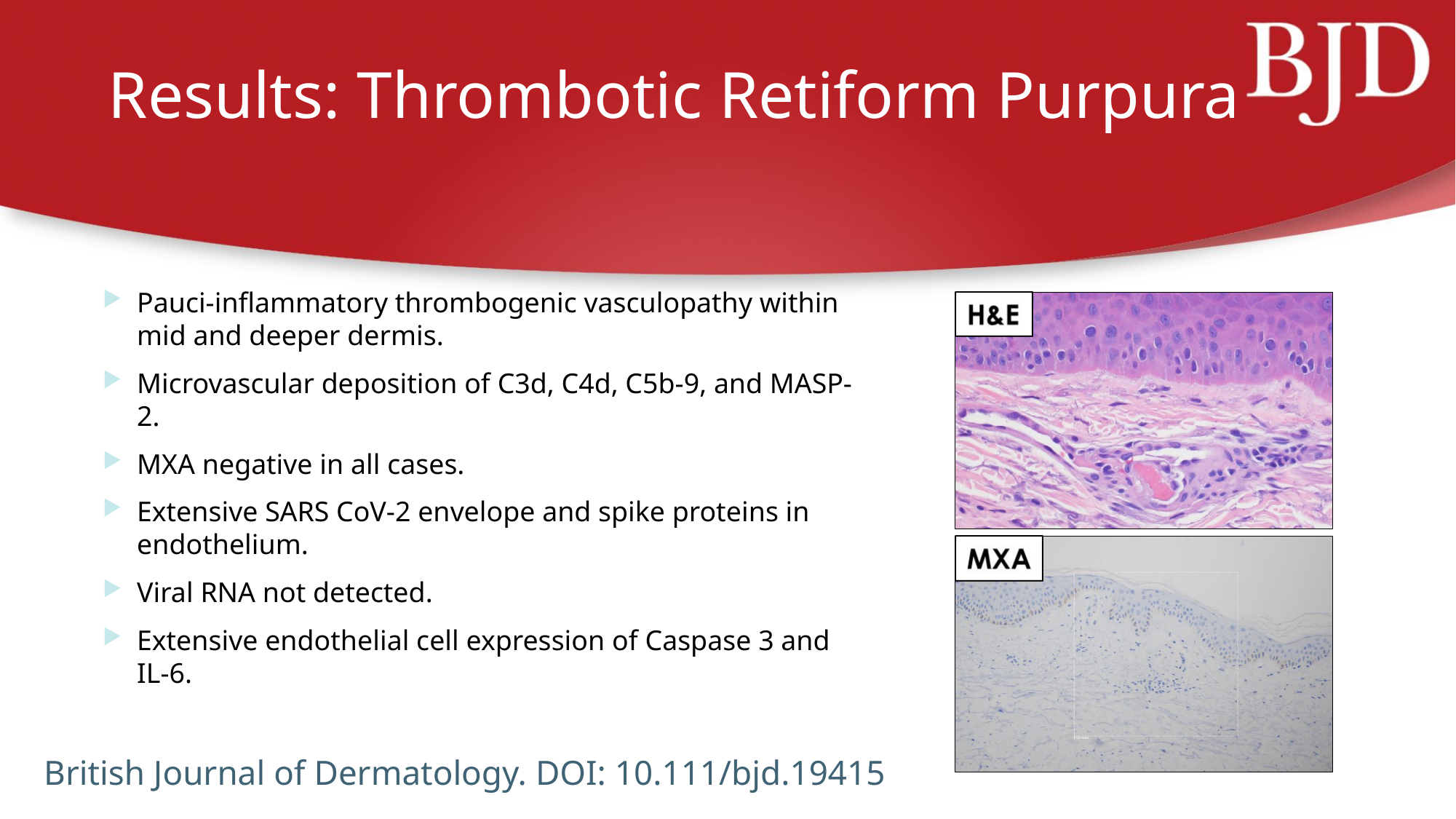

Results: Thrombotic Retiform Purpura
Pauci-inflammatory thrombogenic vasculopathy within mid and deeper dermis.
Microvascular deposition of C3d, C4d, C5b-9, and MASP-2.
MXA negative in all cases.
Extensive SARS CoV-2 envelope and spike proteins in endothelium.
Viral RNA not detected.
Extensive endothelial cell expression of Caspase 3 and IL-6.
British Journal of Dermatology. DOI: 10.111/bjd.19415

## Slide 18
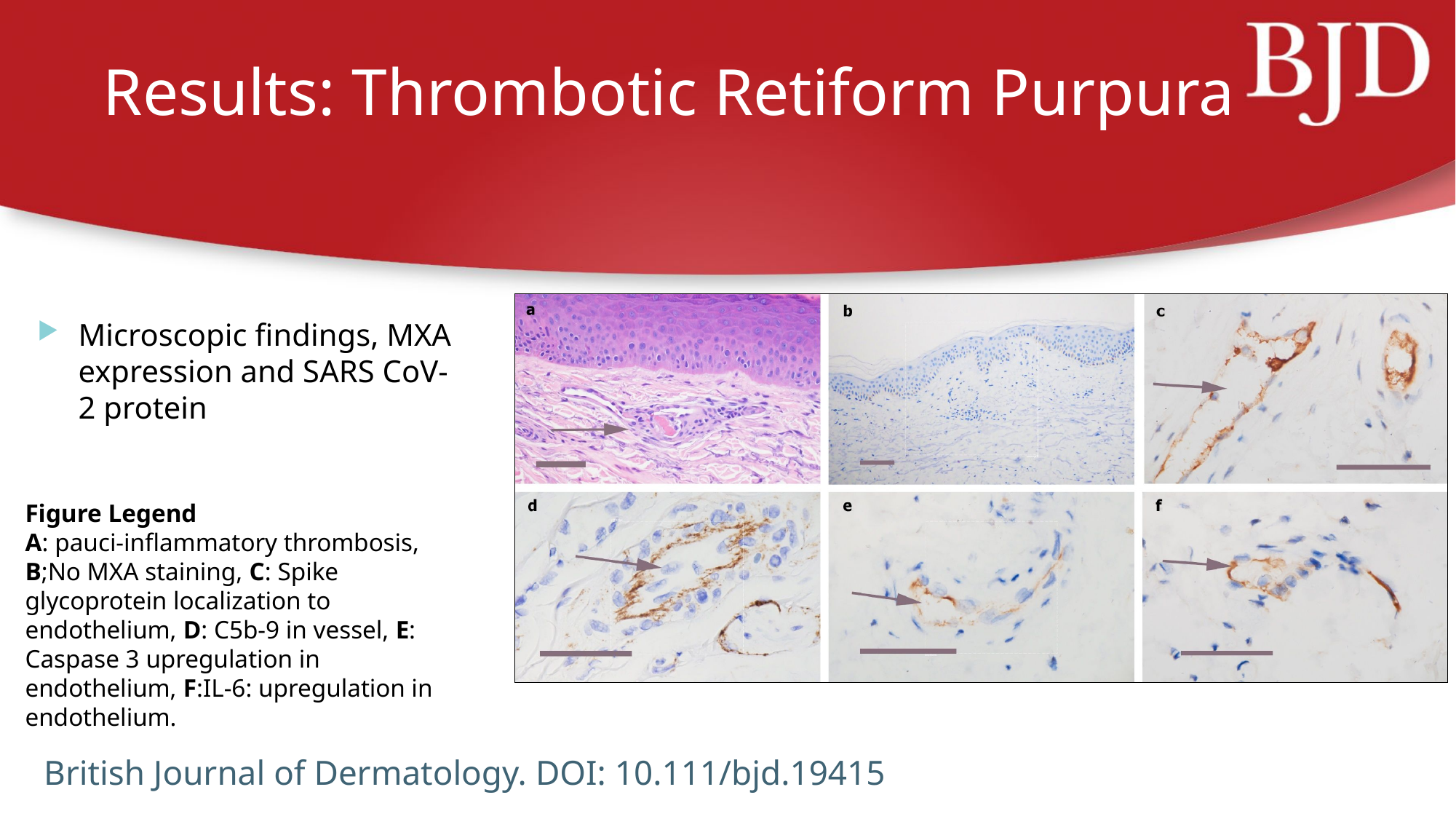

Results: Thrombotic Retiform Purpura
Microscopic findings, MXA expression and SARS CoV-2 protein
Figure Legend
A: pauci-inflammatory thrombosis, B;No MXA staining, C: Spike glycoprotein localization to endothelium, D: C5b-9 in vessel, E: Caspase 3 upregulation in endothelium, F:IL-6: upregulation in endothelium.
British Journal of Dermatology. DOI: 10.111/bjd.19415

## Slide 19
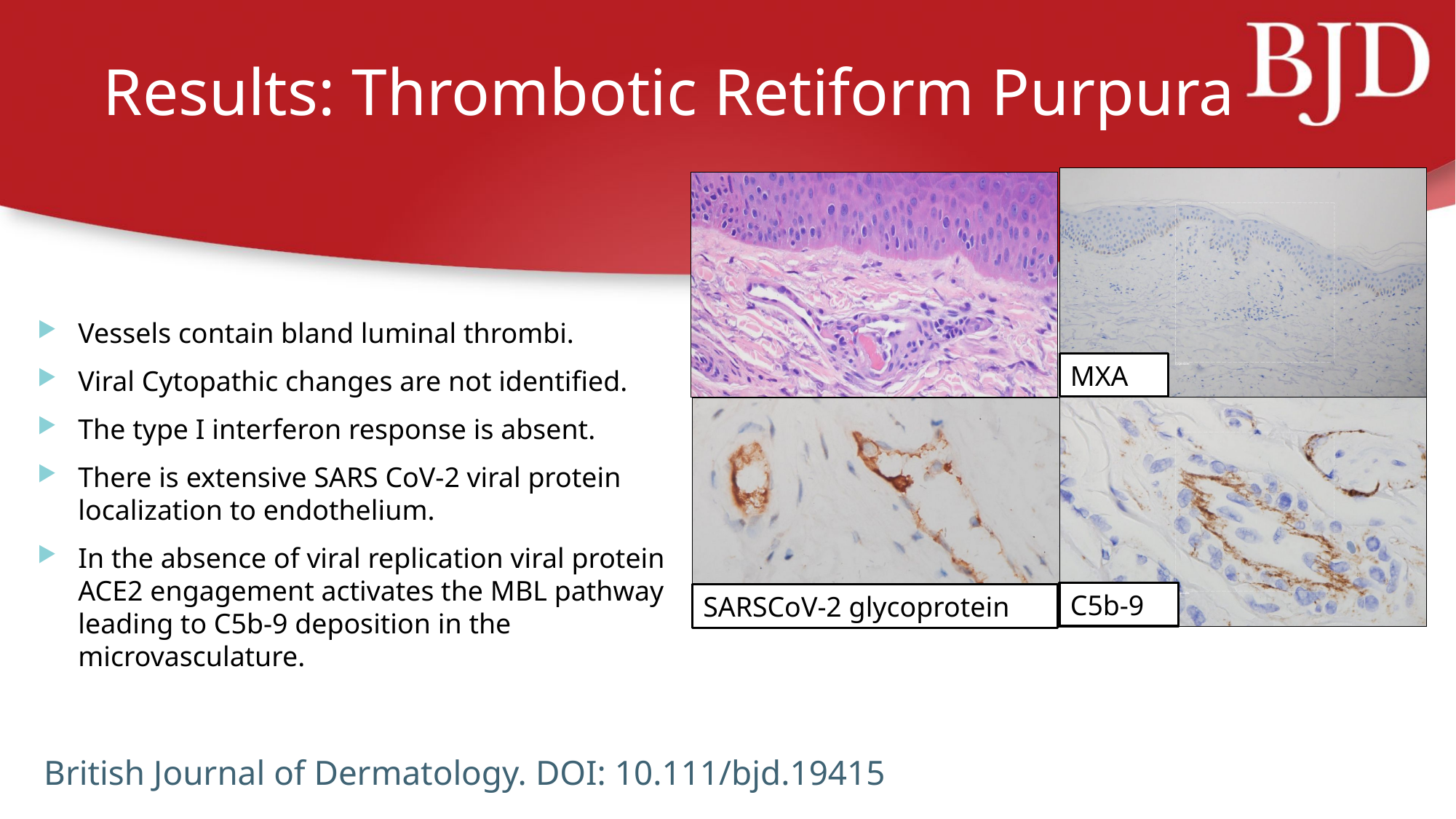

Results: Thrombotic Retiform Purpura
Vessels contain bland luminal thrombi.
Viral Cytopathic changes are not identified.
The type I interferon response is absent.
There is extensive SARS CoV-2 viral protein localization to endothelium.
In the absence of viral replication viral protein ACE2 engagement activates the MBL pathway leading to C5b-9 deposition in the microvasculature.
MXA
C5b-9
SARSCoV-2 glycoprotein
British Journal of Dermatology. DOI: 10.111/bjd.19415

## Slide 20
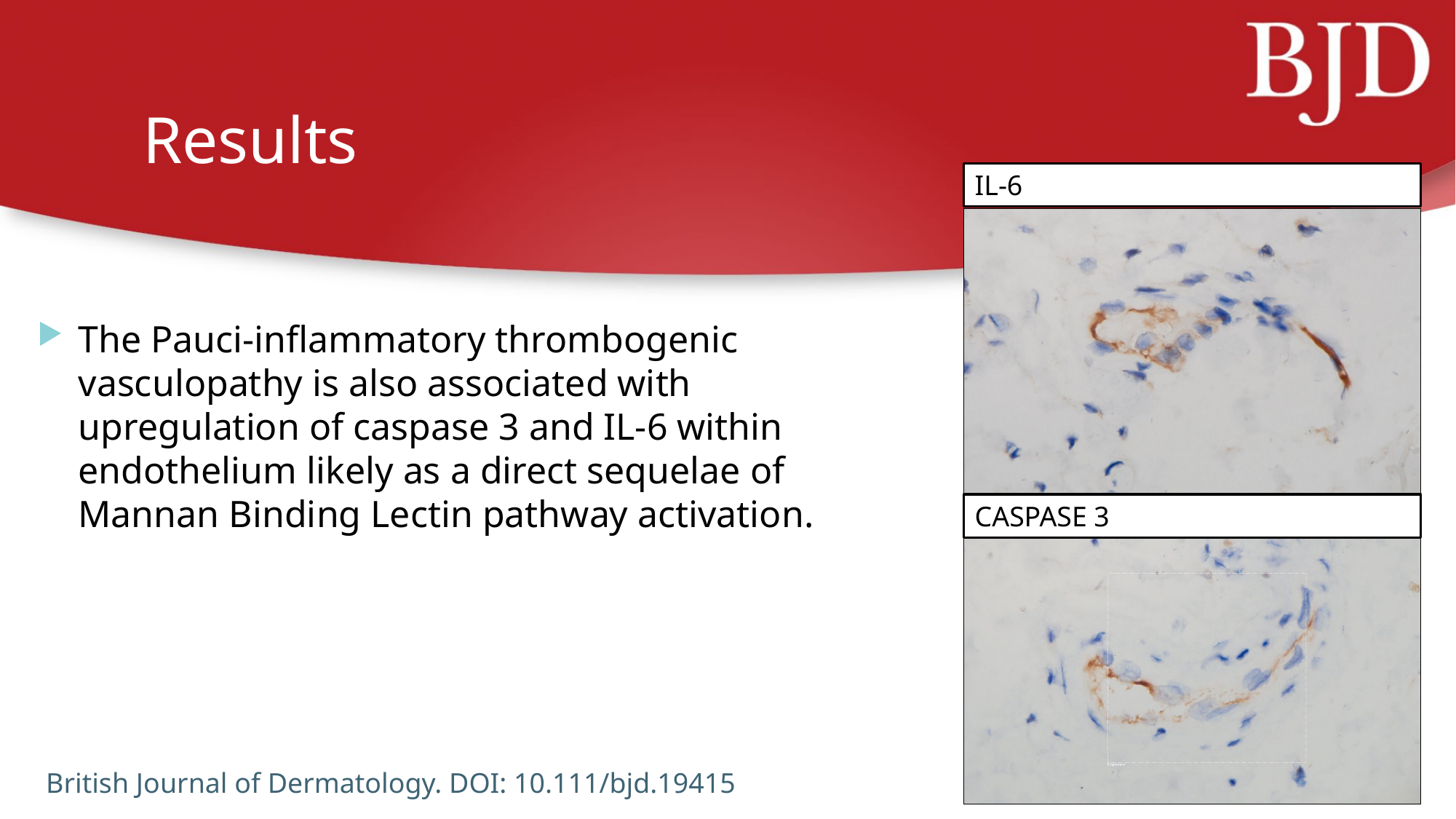

# Results
IL-6
The Pauci-inflammatory thrombogenic vasculopathy is also associated with upregulation of caspase 3 and IL-6 within endothelium likely as a direct sequelae of Mannan Binding Lectin pathway activation.
CASPASE 3
British Journal of Dermatology. DOI: 10.111/bjd.19415

## Slide 21
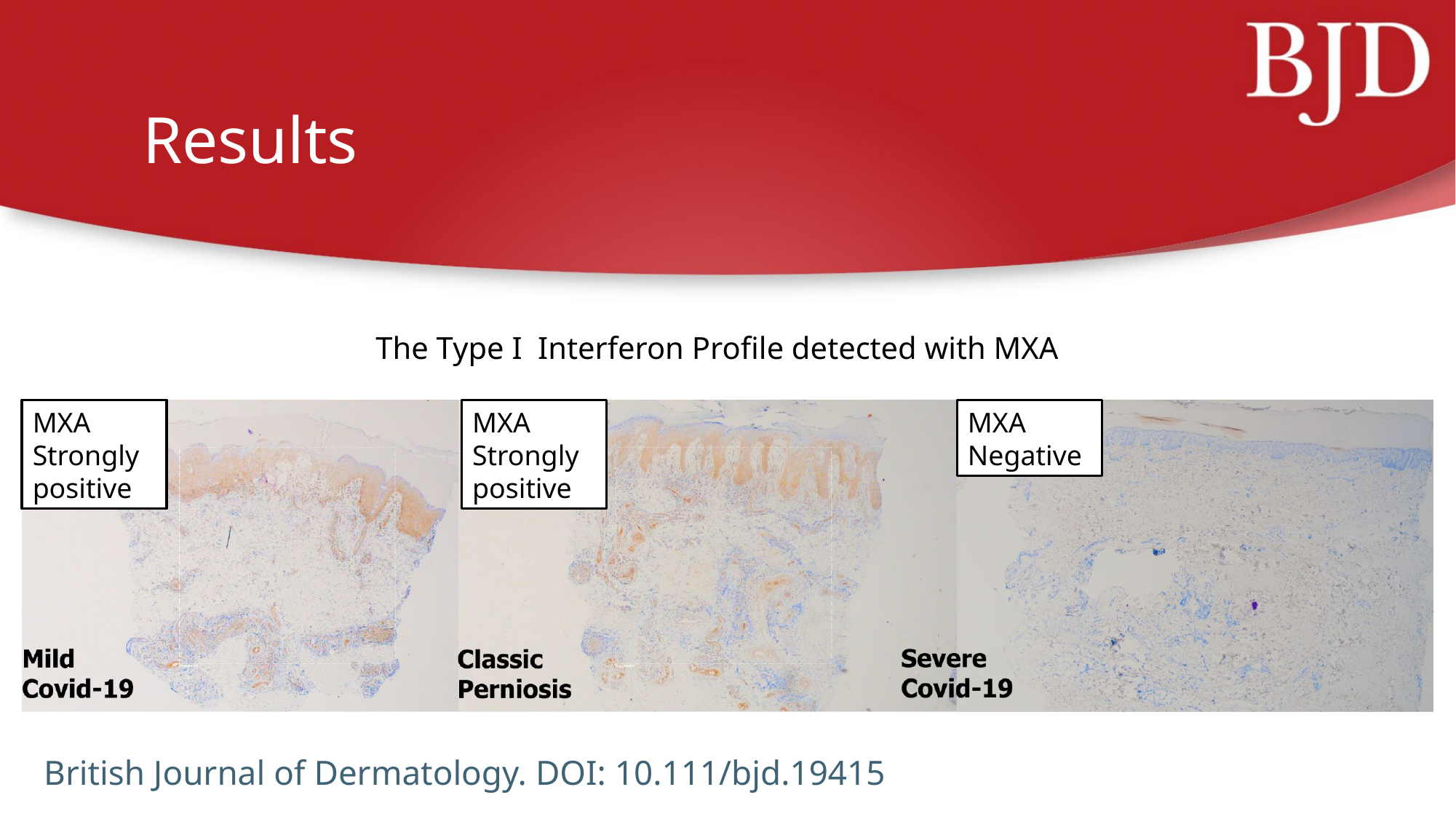

# Results
The Type I Interferon Profile detected with MXA
MXA
Negative
MXA
Strongly
positive
MXA
Strongly
positive
British Journal of Dermatology. DOI: 10.111/bjd.19415

## Slide 22
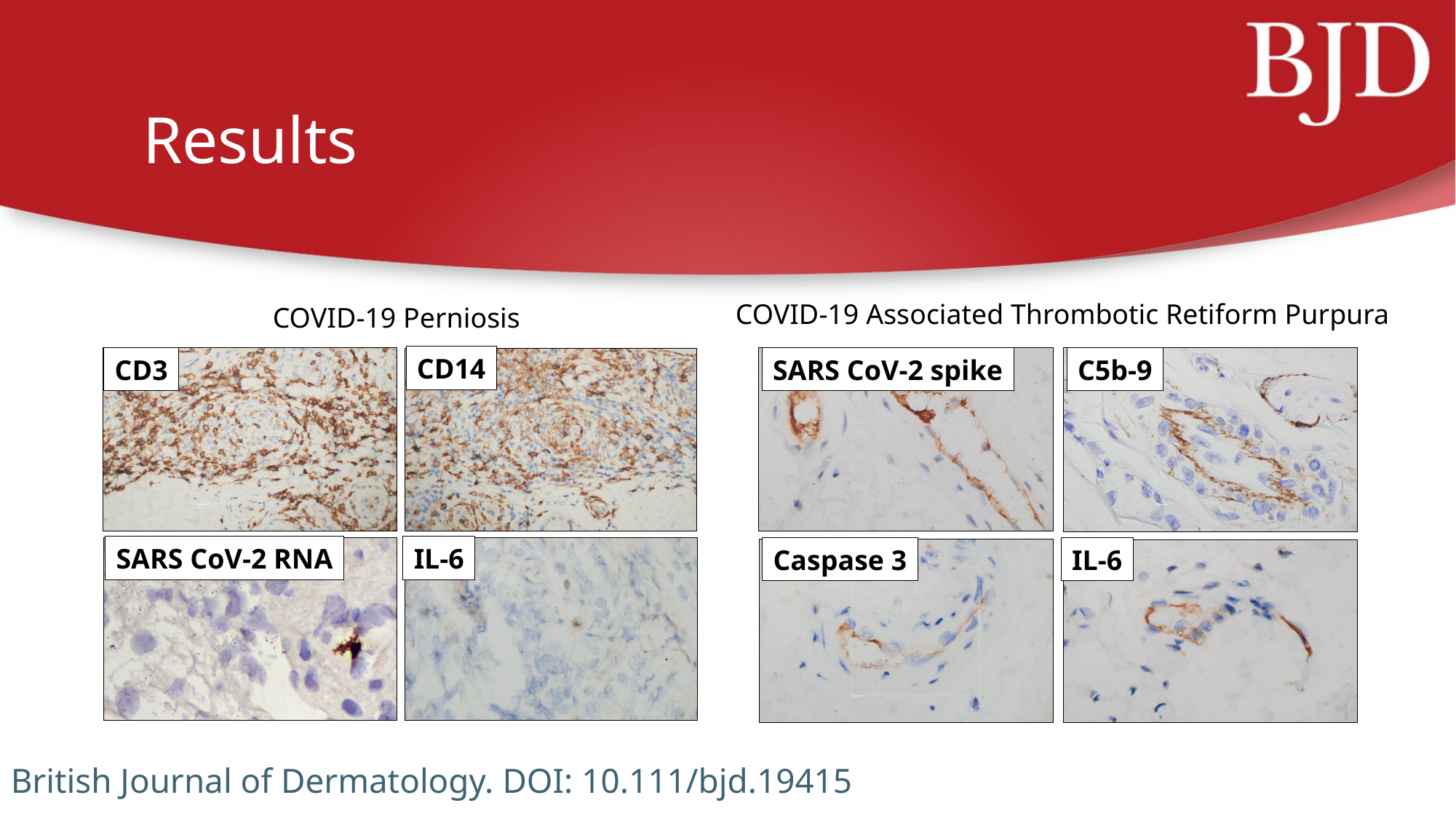

# Results
COVID-19 Associated Thrombotic Retiform Purpura
COVID-19 Perniosis
CD14
CD3
SARS CoV-2 spike
C5b-9
SARS CoV-2 RNA
IL-6
Caspase 3
IL-6
British Journal of Dermatology. DOI: 10.111/bjd.19415

## Slide 23
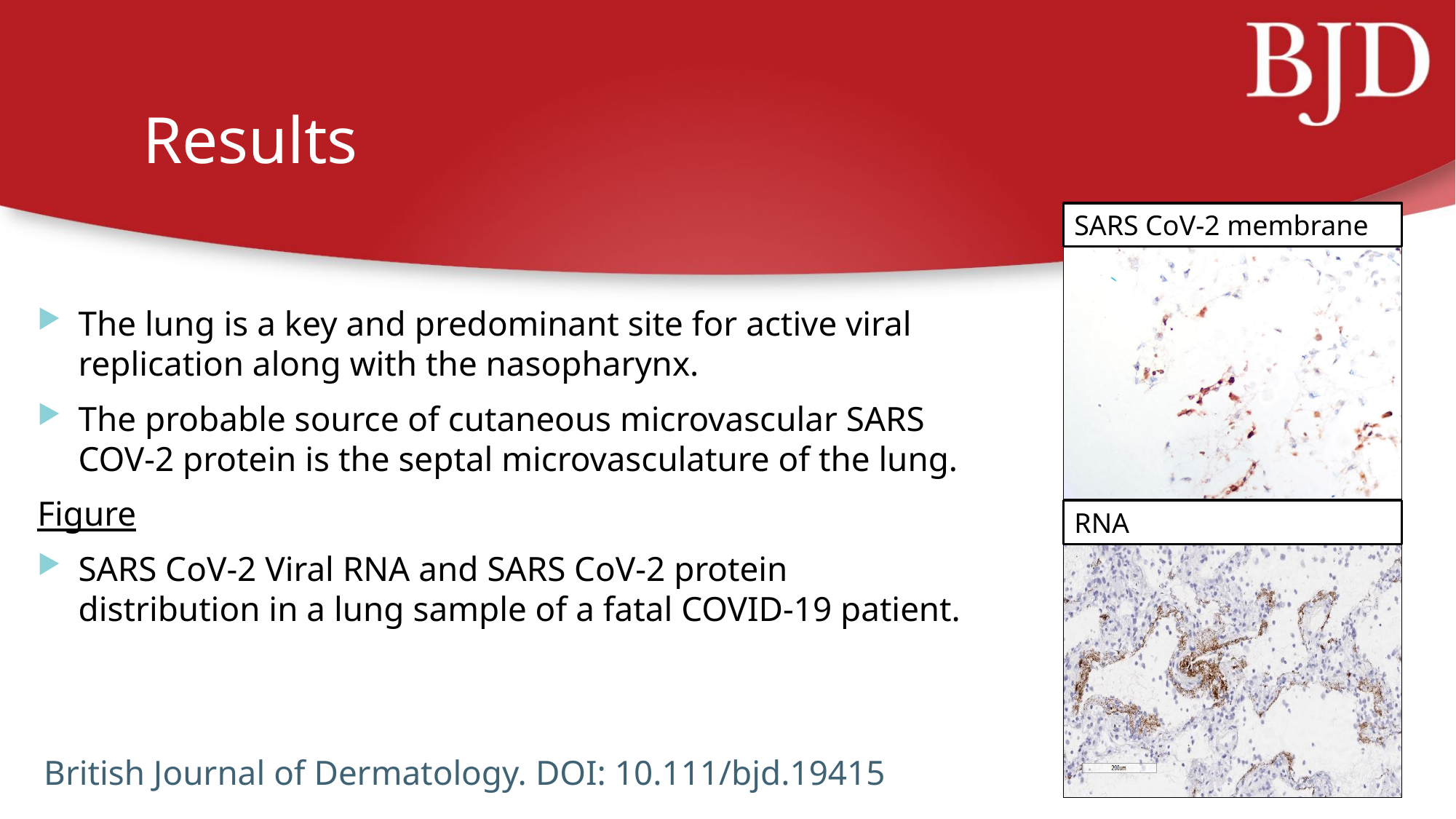

# Results
SARS CoV-2 membrane
The lung is a key and predominant site for active viral replication along with the nasopharynx.
The probable source of cutaneous microvascular SARS COV-2 protein is the septal microvasculature of the lung.
Figure
SARS CoV-2 Viral RNA and SARS CoV-2 protein distribution in a lung sample of a fatal COVID-19 patient.
RNA
British Journal of Dermatology. DOI: 10.111/bjd.19415

## Slide 24
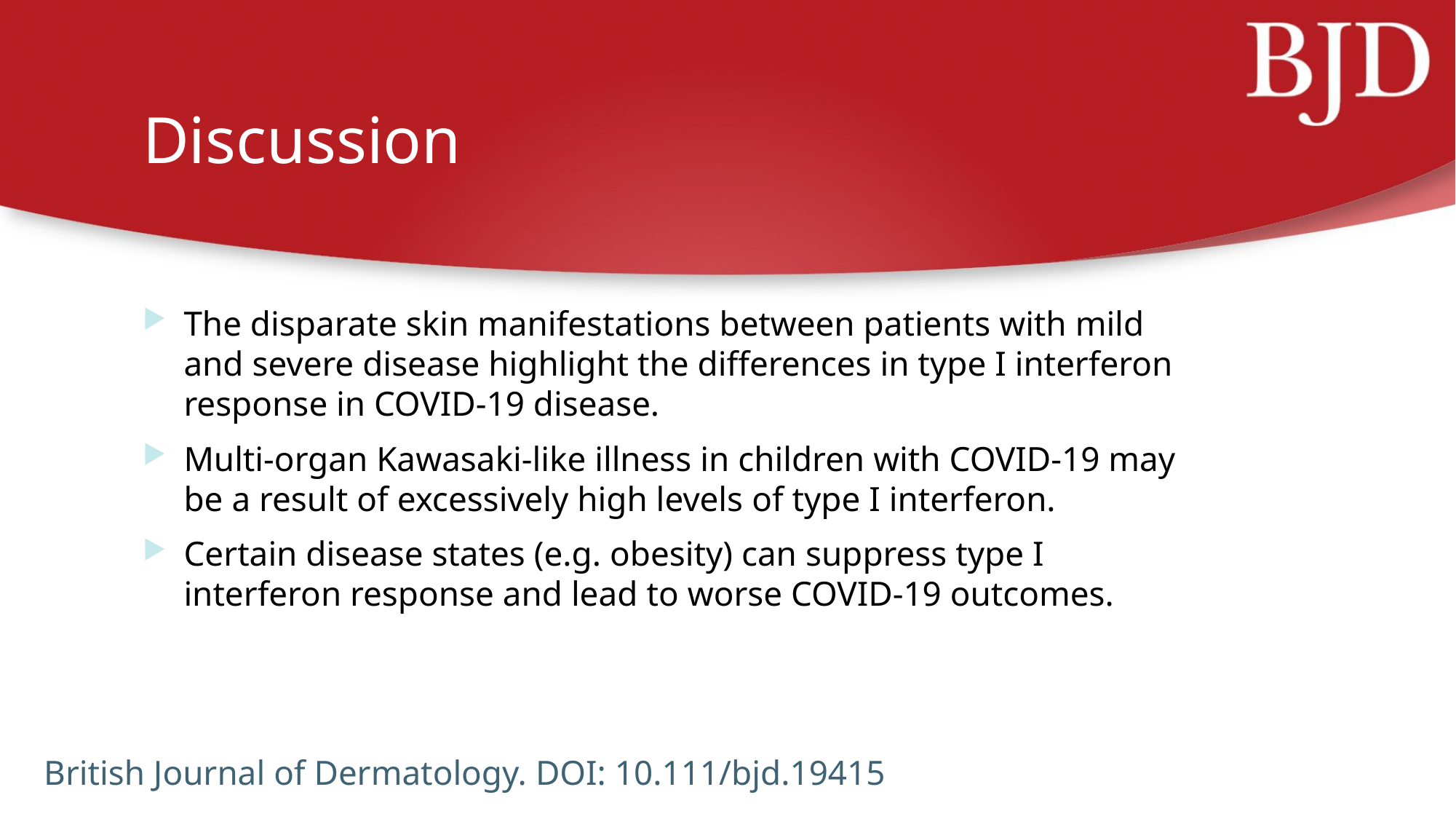

# Discussion
The disparate skin manifestations between patients with mild and severe disease highlight the differences in type I interferon response in COVID-19 disease.
Multi-organ Kawasaki-like illness in children with COVID-19 may be a result of excessively high levels of type I interferon.
Certain disease states (e.g. obesity) can suppress type I interferon response and lead to worse COVID-19 outcomes.
British Journal of Dermatology. DOI: 10.111/bjd.19415

## Slide 25
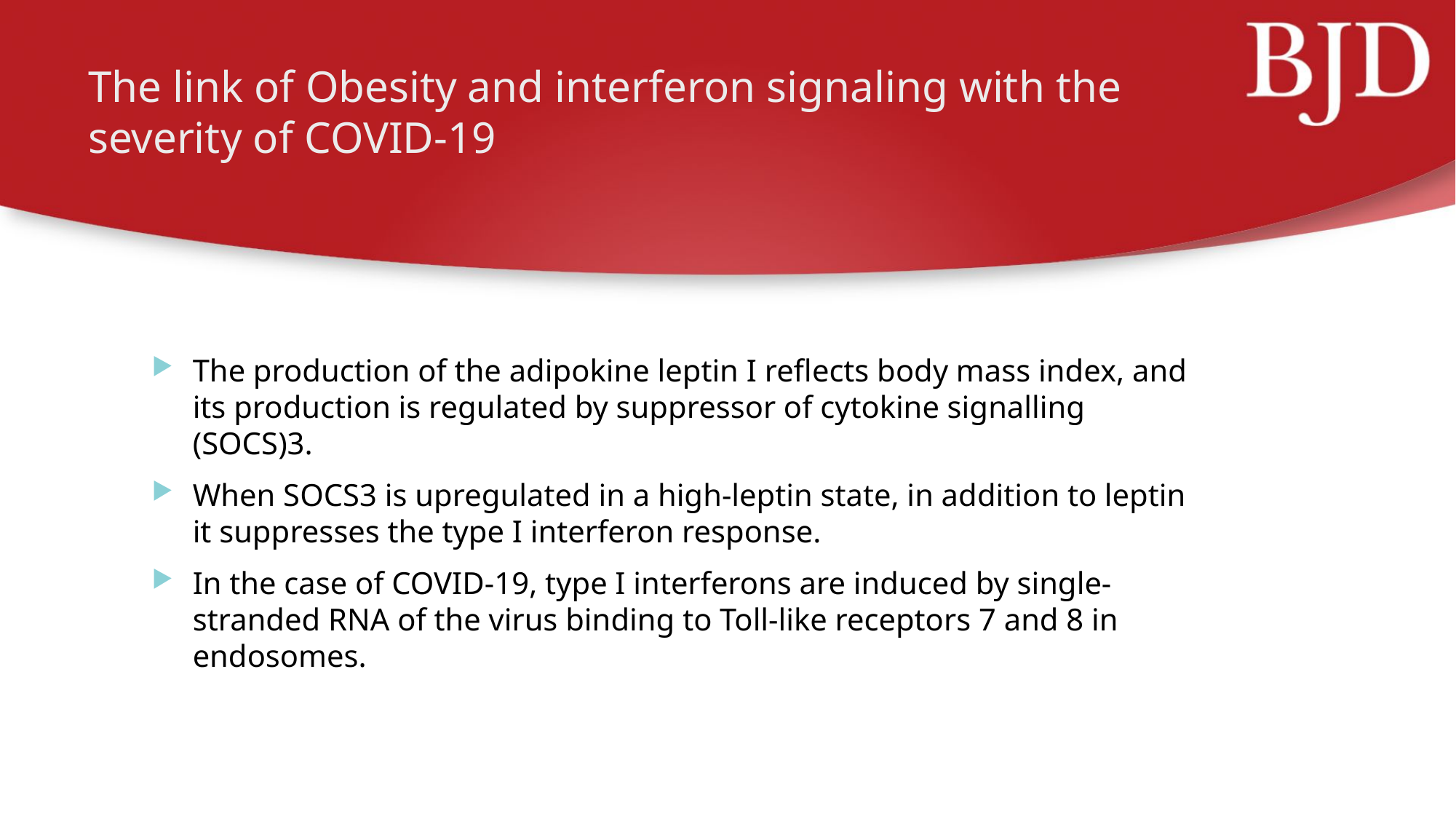

# The link of Obesity and interferon signaling with the severity of COVID-19
The production of the adipokine leptin I reflects body mass index, and its production is regulated by suppressor of cytokine signalling (SOCS)3.
When SOCS3 is upregulated in a high-leptin state, in addition to leptin it suppresses the type I interferon response.
In the case of COVID-19, type I interferons are induced by single- stranded RNA of the virus binding to Toll-like receptors 7 and 8 in endosomes.

## Slide 26
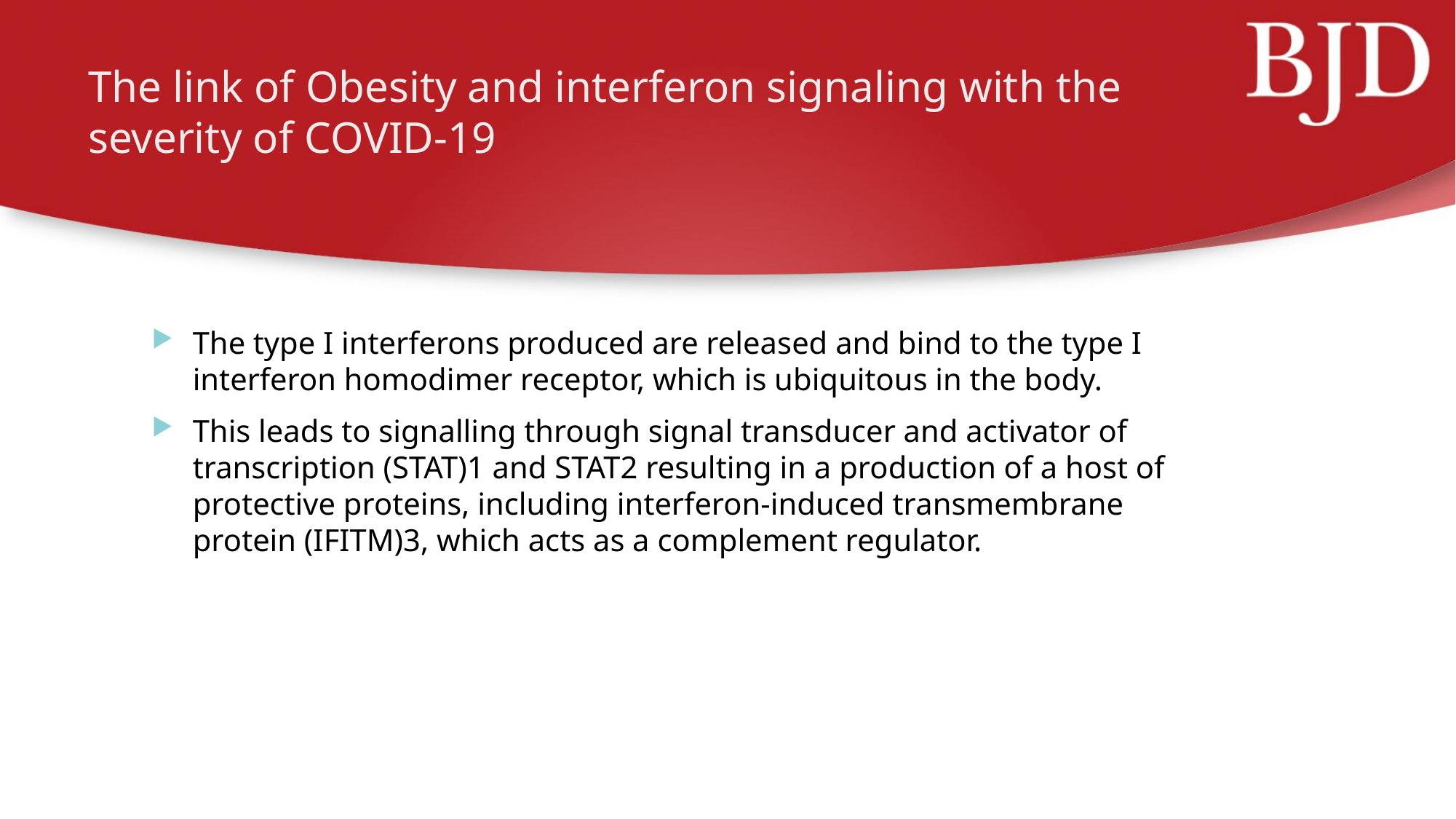

# The link of Obesity and interferon signaling with the severity of COVID-19
The type I interferons produced are released and bind to the type I interferon homodimer receptor, which is ubiquitous in the body.
This leads to signalling through signal transducer and activator of transcription (STAT)1 and STAT2 resulting in a production of a host of protective proteins, including interferon-induced transmembrane protein (IFITM)3, which acts as a complement regulator.

## Slide 27
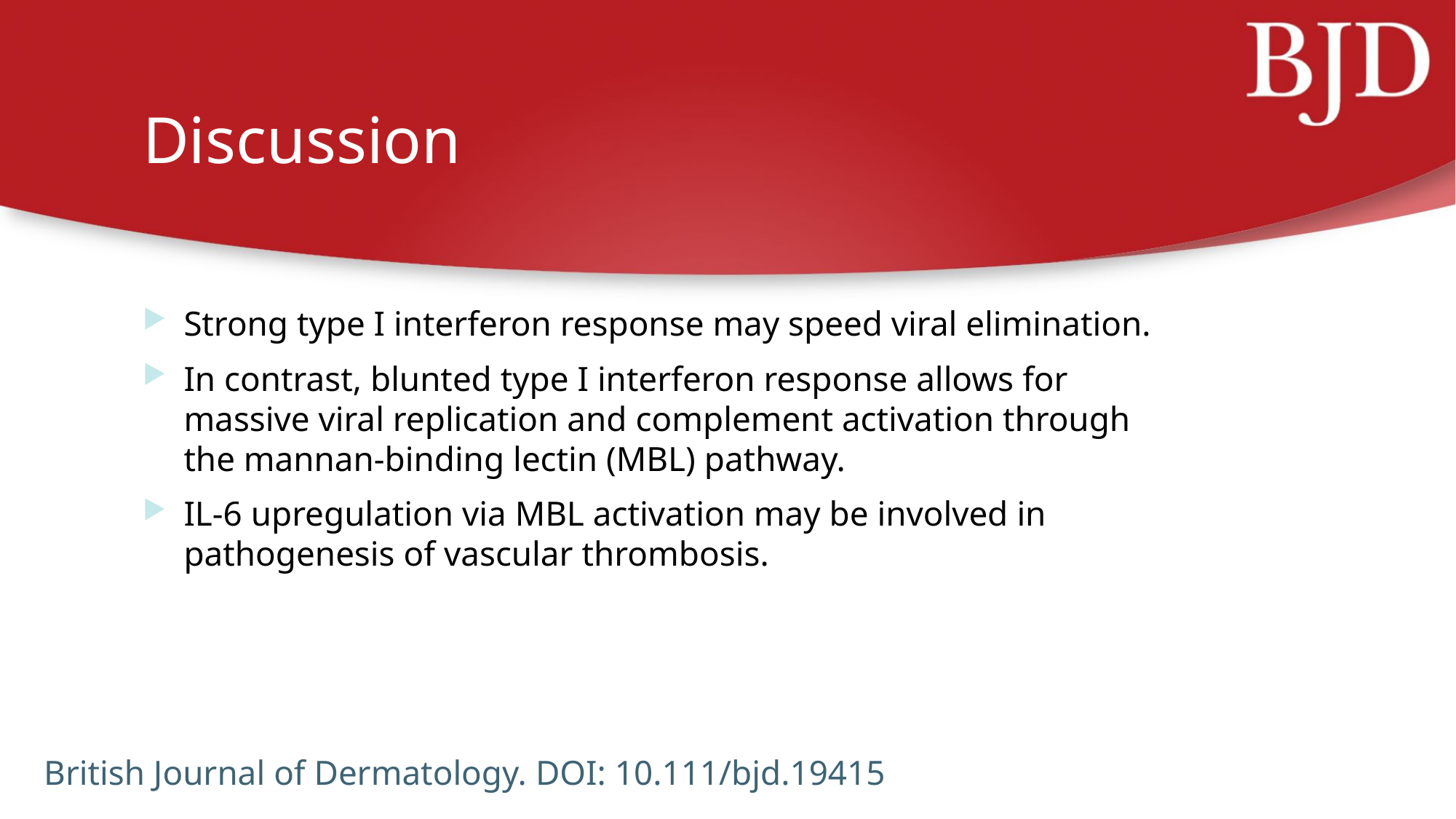

# Discussion
Strong type I interferon response may speed viral elimination.
In contrast, blunted type I interferon response allows for massive viral replication and complement activation through the mannan-binding lectin (MBL) pathway.
IL-6 upregulation via MBL activation may be involved in pathogenesis of vascular thrombosis.
British Journal of Dermatology. DOI: 10.111/bjd.19415

## Slide 28
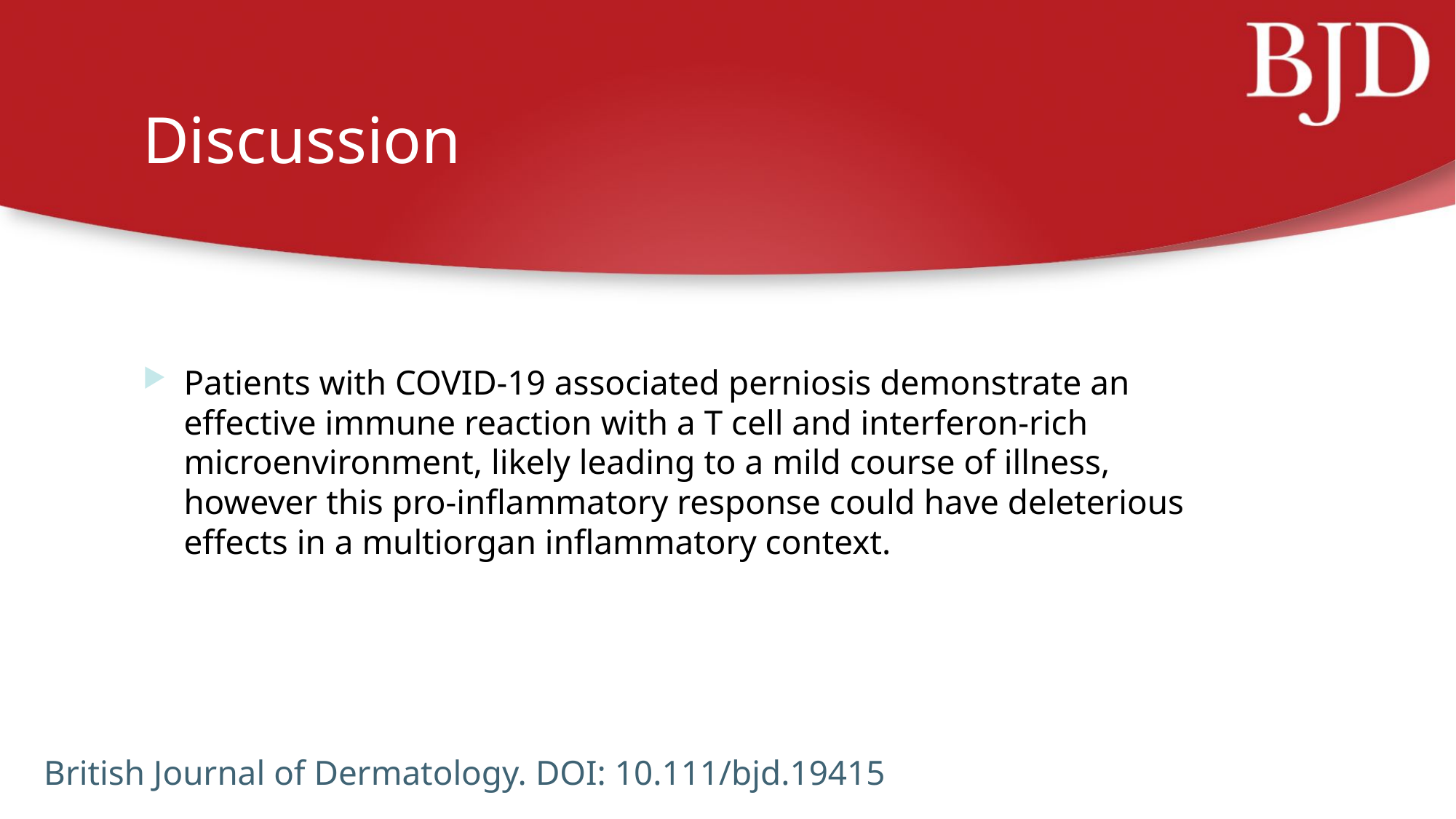

# Discussion
Patients with COVID-19 associated perniosis demonstrate an effective immune reaction with a T cell and interferon-rich microenvironment, likely leading to a mild course of illness, however this pro-inflammatory response could have deleterious effects in a multiorgan inflammatory context.
British Journal of Dermatology. DOI: 10.111/bjd.19415

## Slide 29
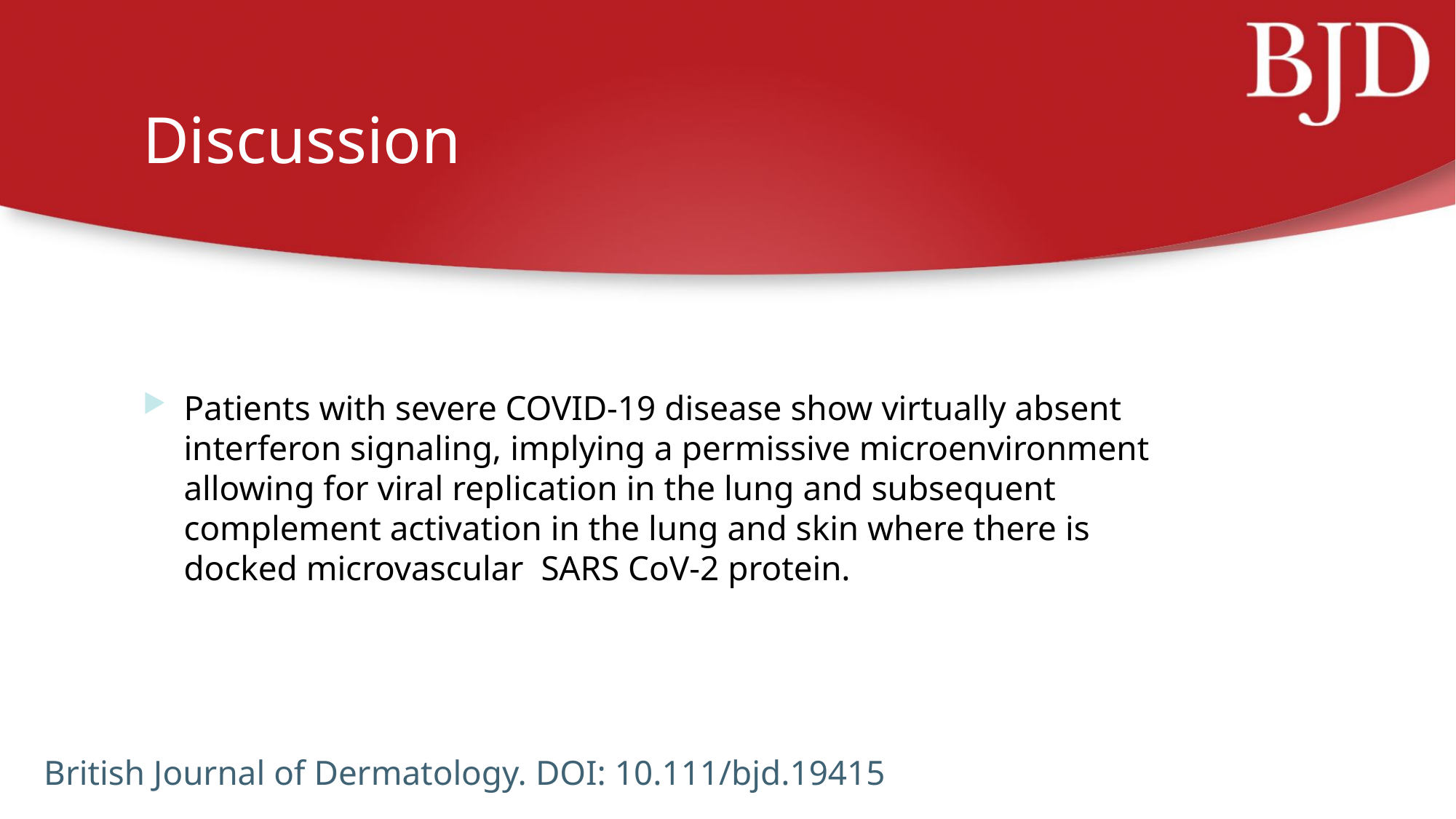

# Discussion
Patients with severe COVID-19 disease show virtually absent interferon signaling, implying a permissive microenvironment allowing for viral replication in the lung and subsequent complement activation in the lung and skin where there is docked microvascular SARS CoV-2 protein.
British Journal of Dermatology. DOI: 10.111/bjd.19415

## Slide 30
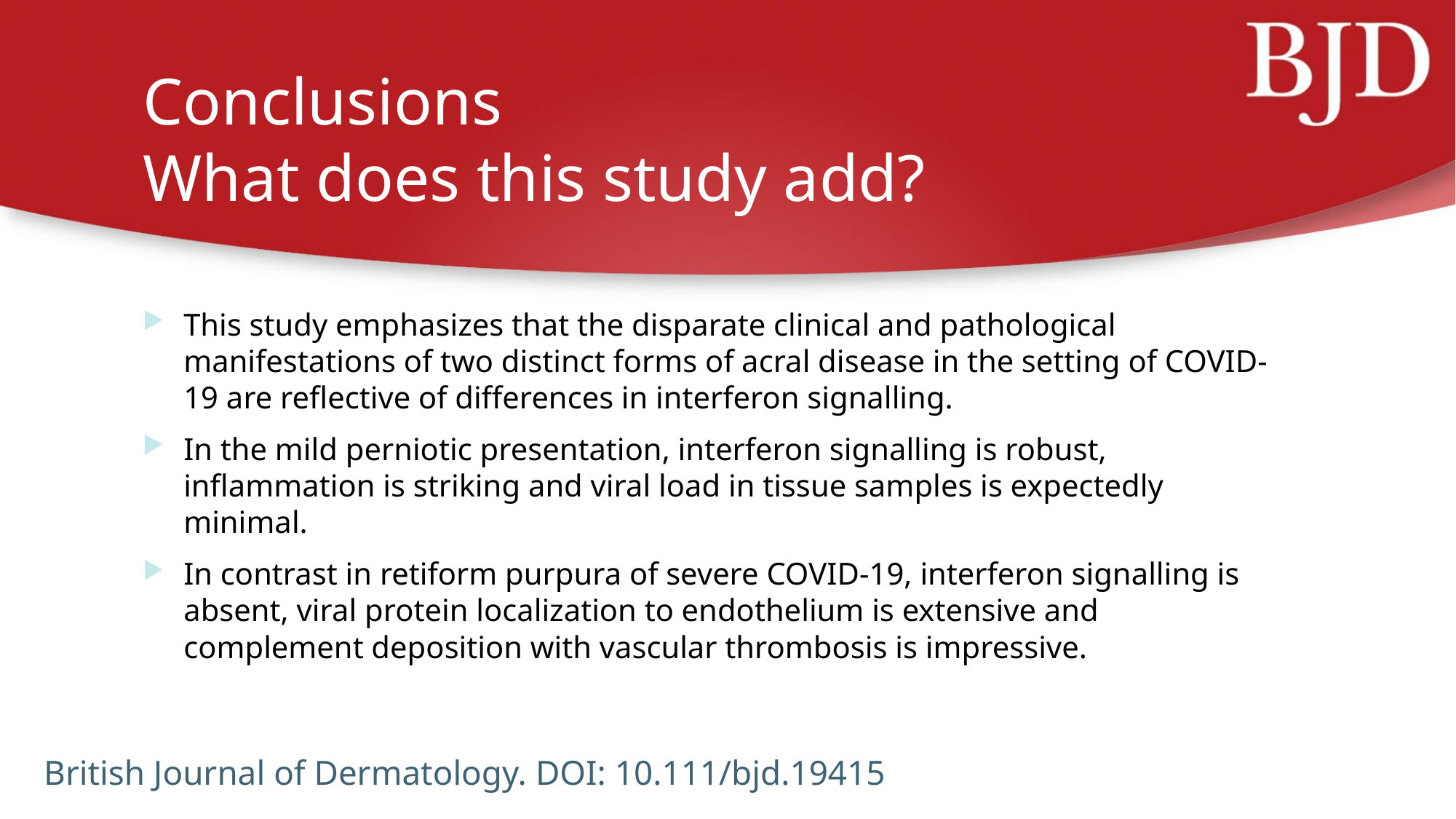

# Conclusions What does this study add?
This study emphasizes that the disparate clinical and pathological manifestations of two distinct forms of acral disease in the setting of COVID-19 are reflective of differences in interferon signalling.
In the mild perniotic presentation, interferon signalling is robust, inflammation is striking and viral load in tissue samples is expectedly minimal.
In contrast in retiform purpura of severe COVID-19, interferon signalling is absent, viral protein localization to endothelium is extensive and complement deposition with vascular thrombosis is impressive.
British Journal of Dermatology. DOI: 10.111/bjd.19415

## Slide 31
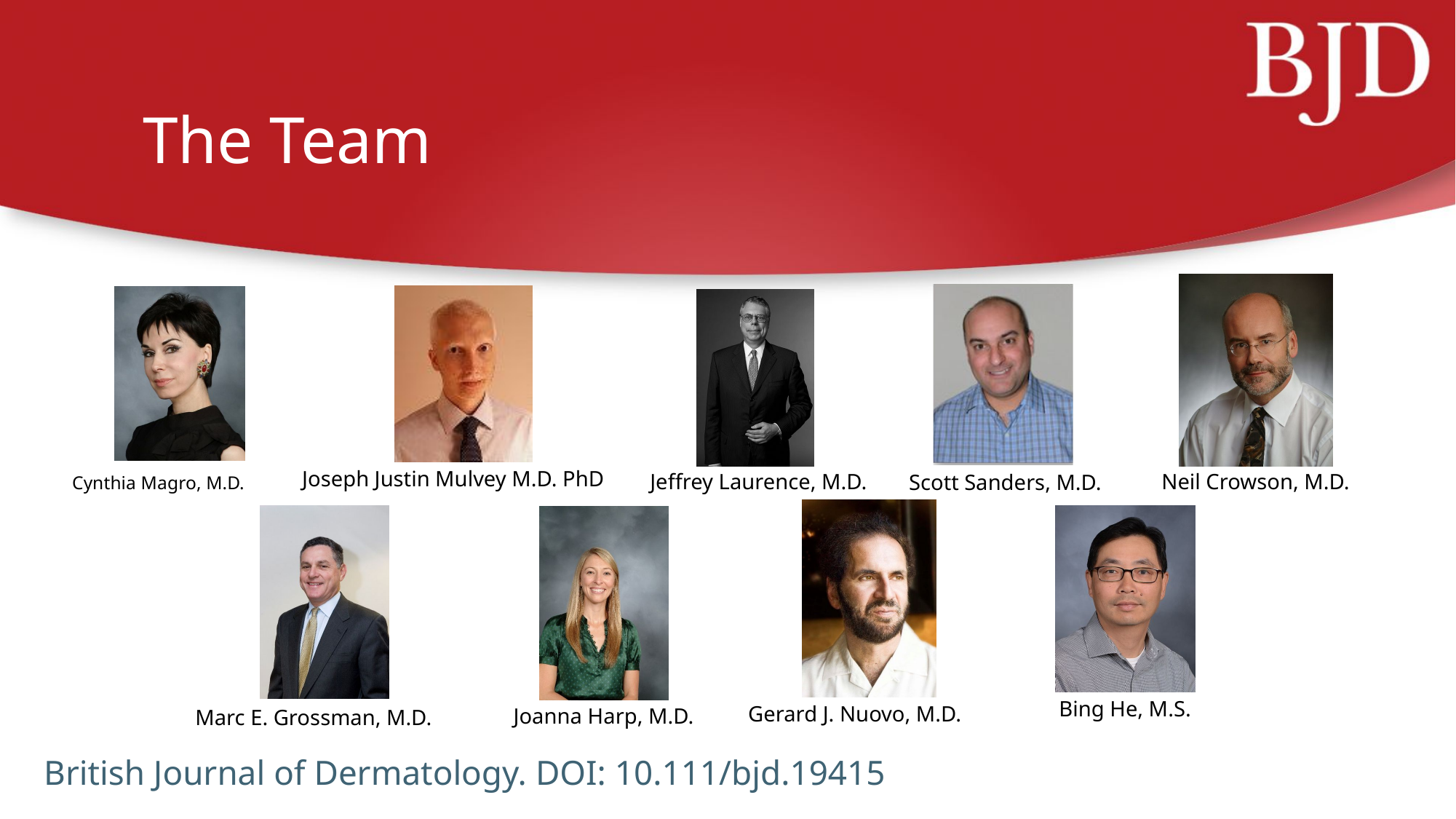

# The Team
Joseph Justin Mulvey M.D. PhD
Jeffrey Laurence, M.D.
Neil Crowson, M.D.
Scott Sanders, M.D.
Cynthia Magro, M.D.
Bing He, M.S.
Gerard J. Nuovo, M.D.
Joanna Harp, M.D.
Marc E. Grossman, M.D.
British Journal of Dermatology. DOI: 10.111/bjd.19415

## Slide 32
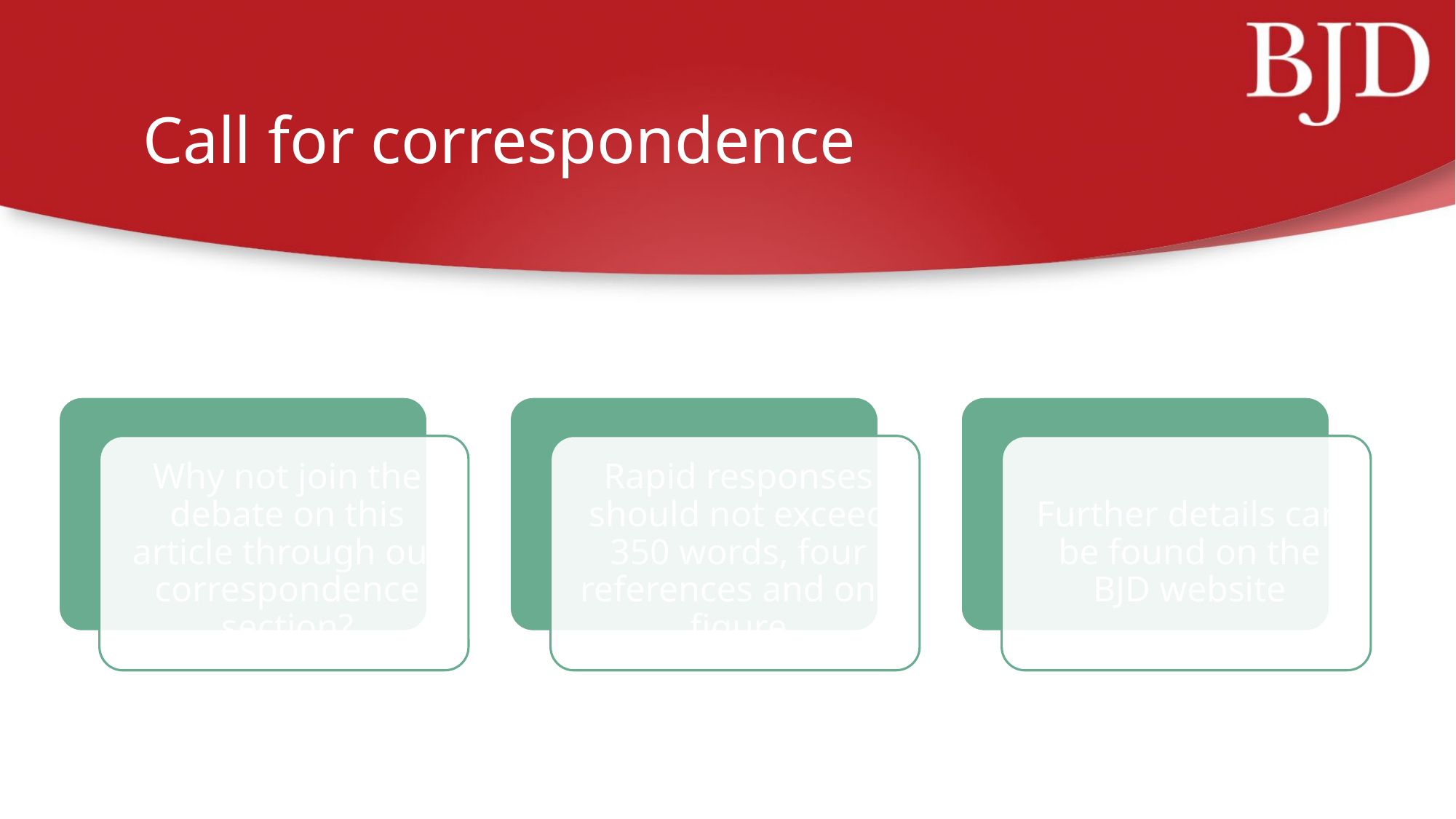

# Call for correspondence
